# Supplementary material for: Quantifying pupil-to-pupil SARS-CoV-2 transmission and the impact of lateral flow testing in English secondary schools
Source: Nat Commun. 2022 Mar 1;13:1106. doi: 10.1038/s41467-022-28731-9 (PMC8888696; doi:10.1038/s41467-022-28731-9)
Supplement: Supplementary file 1 — Supplementary Information [file 41467_2022_28731_MOESM1_ESM.pdf]

# Quantifying pupil-to-pupil SARS-CoV-2 transmission and the impact of lateral flow testing in English secondary schools Supplementary Material

Trystan Leng[1,2*], Edward M. Hill[1,2], Alex Holmes[1,3], Emma Southall[1,3], Robin N. Thompson[1,2], Michael J. Tildesley[1,2] Matt J. Keeling[1,2], Louise Dyson[1,2]

**1.** The Zeeman Institute for Systems Biology & Infectious Disease Epidemiology Research, School of Life Sciences and Mathematics Institute, University of Warwick, Coventry, CV4 7AL, United Kingdom

**2.** JUNIPER – Joint UNIversities Pandemic and Epidemiological Research, https://maths.org/juniper/

**3.** Mathematics for Real-World Systems Centre for Doctoral Training, University of Warwick, Coventry, CV4 7AL. United Kingdom

*Corresponding Author: trystan.leng@warwick.ac.uk

# Supplementary Text 1 Data sources used in this study

## 1.1 Community swab testing

SARS-CoV-2 testing data have been collected and recorded daily since the virus was first detected in the UK[1]. Pillar 2 data refers to community swab testing data from those who have sought PCR tests due to COVID-19 symptoms, or from those taking a lateral flow device test (LFT). We fit our model to two strands of Pillar 2 data: (i) the proportion of 11-16 year olds testing positive in a PCR test each week (excluding confirmatory PCR tests, which are accounted for in LFT testing data) from 31st August 2020, and (ii) the proportion of 11-16 year olds testing positive to an LFT test each day from 8th March 2021. We focus on the 11-16 year olds age group as the vast majority of 11-16 year olds attend secondary schools in England; we therefore view that these data can be used as a proxy for testing rates within secondary school pupils. We excluded 17-18 year olds as some of these will be employed or attending university, and therefore trends may emerge in testing data in these age groups that do not reflect the dynamics within secondary schools, but rather reflect dynamics in different settings (e.g. universities). We also used Pillar 2 data to inform the level of LFT participation in each LTLA in the model. The participation in each LTLA on a given day was taken as the proportion of 10-19 year olds in that LTLA who recorded either a positive or negative LFT on that day. We used the broader age range of 10-19 year olds here due to the negative Pillar 2 data only being available in five-year age bands. It is likely that a considerable proportion of negative home tests remain unrecorded; to account for this, we fit the level of underreporting of negative tests, which scales up the 'true' number of negative LFT tests and hence increases participation levels.

## 1.2 S-gene data

The Alpha (B.1.1.7) variant is characterised by a deletion in the genome at site 69-70 associated with the spike protein; this leads to the ThermoFisher TaqPath quantitative PCR assay, used as the main diagnostic tool in many regions of England, failing to amplify the S-gene target. As such, infections that are confirmed by PCR but where the S-gene is not detected (often termed as 'S-gene failures') provide a key indicator of the geographical spread of the Alpha variant. We therefore used the change in proportion of S-gene failures within an LTLA as a proxy for the sigmoidal growth of the Alpha variant which is associated with higher transmission rates than the original wild-type virus.

## 1.3 Population by LTLA

We obtained the population sizes in each LTLA from the Office for National Statistics population estimates, using the mid-year estimates from 2019[2]. We assumed that 11-16 year olds who tested positive in a particular

LTLA also attended school in that LTLA.

### 1.4 School absence data

Since September 2020, secondary schools have recorded data regarding absences and confirmed COVID-19 cases, available through the Department for Education: Educational Setting Status data[3]. In particular, each school recorded the number of absences due to confirmed COVID-19 cases among pupils each day. While these data are insufficient to establish the total number of cases in any particular school (as each case is absent for multiple days), one can obtain the peak number of confirmed cases in each school. To capture the heterogeneity in outbreak sizes between schools, we fitted our model to the distributions of peak number of confirmed cases in schools from September to December 2020 and from March to May 2021. Secondary schools were closed to the majority of pupils in January and February 2021 as part of a national lockdown, and because of these closures we did not consider confirmed cases in schools during this period.

In addition to the number of absences due to confirmed cases of COVID-19, the number of absences due to other within-school COVID-19 related reasons were also recorded in the data. Each absence was either due to being a confirmed COVID-19 case, being a suspected COVID-19 case, or having potentially had contact with a COVID-19 case from inside the educational setting (after which the student was requested to isolate). Prior to 10th October 2020, the number of pupils that were asked to isolate due to potential contact with a COVID-19 case were not separated according to whether the case was from within or outside the educational setting. As such, the total number of absences for within-school COVID-19 related reasons over this period are overestimated.

## Supplementary Text 2 Constructing modelled secondary schools

We derived from the Department for Education: Educational Setting Status data[3] the number of pupils attending a given modelled secondary school, the LTLA of a modelled secondary school, the urban or rural status of a school, and close contact group sizes for September-December 2020 and for March-May 2021. From 31st August 2020 to December 2020, 2979 secondary schools reported such data, while 2861 secondary schools reported this data from 8th March 2021 to 23rd May 2021. The number of pupils for each secondary school in the data was taken as the median reported number of pupils on roll from September to December 2020. Whether a school included a sixth form was also specified – we assumed schools that had a sixth form to be comprised of seven year groups, while schools without a sixth form were assumed to be comprised of five year groups. This data also contains information regarding whether schools are situated in rural or urban communities. We classified all schools recorded as belonging to an area classification containing the term rural (e.g. 'Rural hamlet and isolated dwellings') as rural schools, and classified all schools recorded belonging to an area classification containing the term urban (e.g. 'Urban major conurbation') as urban schools.

While the size of groups schools send home may vary through time and may be context dependent, we can use absences data from each term to obtain a proxy for the number of close contacts schools are sending home upon identification of a positive case. Specifically, we considered the size of groups different schools sent home after one case by finding the median number of COVID-19 related absences for each school, given there was one confirmed COVID-19 positive pupil (and no confirmed COVID-19 positive teachers). We found close contact group sizes for September-December 2020 and March-May 2021 separately, as schools in general isolated smaller groups of pupils in the latter term.

63.5% of schools recorded a day with exactly one confirmed COVID-19 positive pupils from 31st August 2020 to 18th December 2020, while 54.0% recorded a day with exactly one confirmed COVID-19 positive pupils from 8th March 2021 to 23rd May 2021. Schools without exactly one reported case were similar in their distribution of school sizes to those that did report exactly one case for September-December 2020, (Supplementary Figure 1a) but smaller schools were more likely to have not reported exactly one case from March-May 2021 (Supplementary Figure 1b). Different approaches appear to have been taken by different schools after a confirmed case. In the majority of cases, close contact group sizes do not scale with school size, while other schools appear to have isolated year groups or some fraction of the school, which does scale with school size (Supplementary Figures 1c and 1d). Accordingly, we assumed that close contact group sizes did not scale with school size, and the close contact group sizes for schools that did not report one confirmed COVID-19 positive pupil in each term were sampled from the distribution of reported close contact sizes. We adjusted school sizes, year group sizes, and close contact group sizes for both terms such that year group sizes were a divisor of school sizes, and close contact group sizes for both terms were a divisor of year group sizes. Doing so, we obtained a population of 2979 modelled schools with qualitatively similar

population sizes and implemented close contact isolation policies as reported in Department for Education data (Supplementary Figure 2).

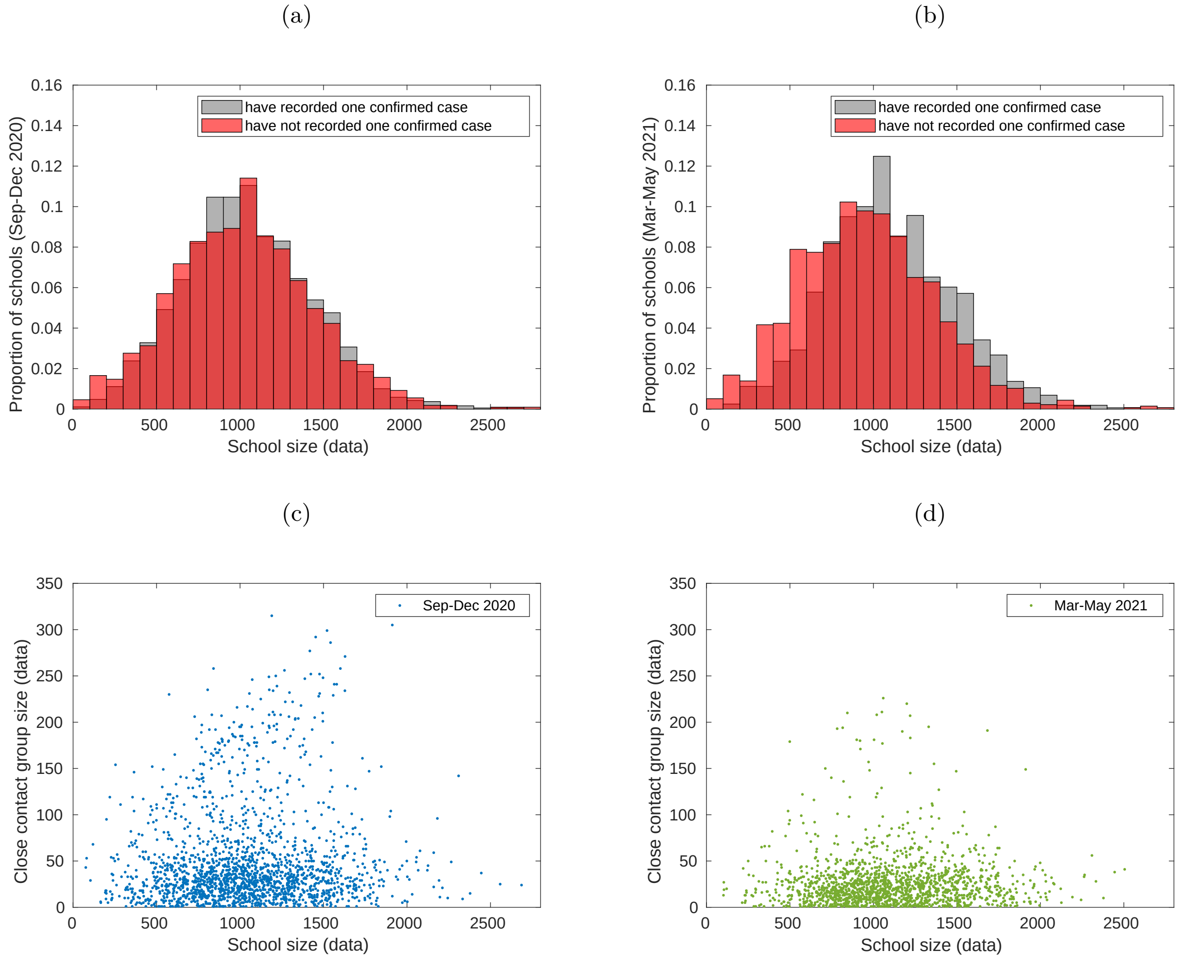


Supplementary Figure 1: **School sizes and close contact group sizes from absences data.** Top panels: Histograms of school sizes for schools who did record (grey) and who did not record (red) at least one day with exactly one confirmed case of COVID-19 from (a) 31st August 2020 to 18th December 2020 and (b) 8th March 2021 to 23rd May 2021. Bottom panels: scatter plot of school sizes against close contact group sizes for (c) 31st August 2020 to 18th December 2020 and (d) 8th March 2021 to 23rd May 2021. School sizes and close contact group sizes were obtained from Department for Education: Education Setting Status data.

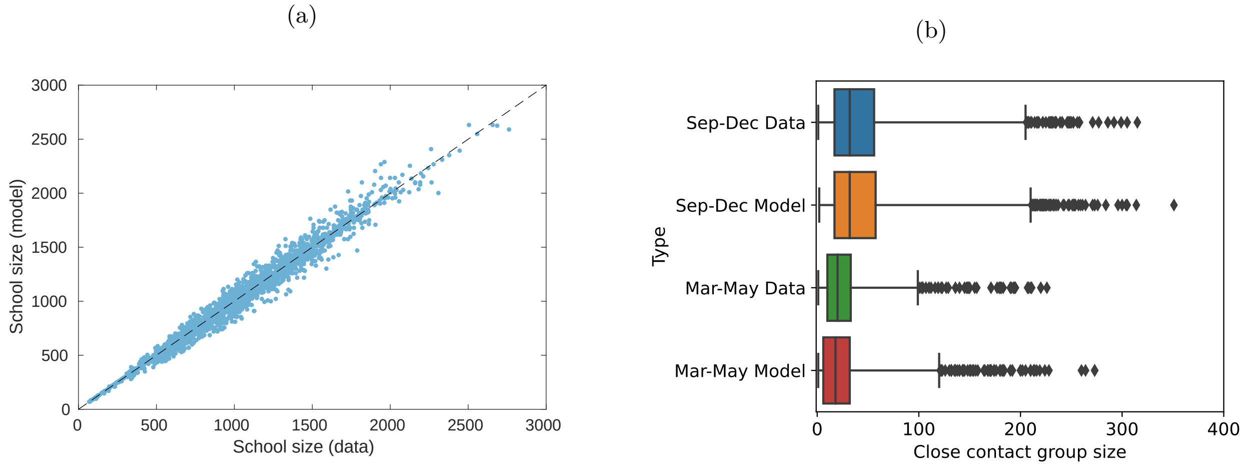


Supplementary Figure 2: **Comparing modelled secondary schools to absences data.** (a) A scatter plot comparing the size of secondary schools obtained from Department for Education: Education Setting Status data, taken as the median reported number of pupils on roll from September to December 2020 for n = 2979 secondary schools reporting data over this period, against modelled secondary school sizes, after adjusting data such that close contact group sizes are divisors of year group sizes and year group sizes are divisors of school sizes. (b) Box plots comparing close contact group sizes inferred from absences data and close contact group sizes used within the model, as a percentage of school size across both terms. Whiskers refer to 2.5% and 97.5% percentiles, edges of boxes refer to 25% and 75% percentiles, and the central lines refer to 50% percentiles. The box plot corresponding to absences data from September-December 2020 was derived from $n$ = 2979 secondary schools who reported data over this period, while for the box plot corresponding to absences data from March-May 2021 was derived from $n$ = 2861 secondary schools who reported data over this period. 2/2979 close contact group sizes above 400 are omitted from the graph for the box plot corresponding to data from September-December 2020.

# Supplementary Text 3 Transmission dynamics in detail

Within the model, a susceptible pupil can become infected via two different routes - they can either be infected by another pupil on a day both pupils are attending school, or they can be infected via external community transmission. On weekends or on days when schools are not open, transmission to pupils occurs only via external infection. Within the model, the probability that an infected pupil attending school infects a susceptible pupil attending school is influenced by a number of factors. The factors determining the expected number of secondary infections from individual $i$ on day $d$ assuming all their contacts are susceptible, denoted $\lambda_i(d)$, are detailed in Supplementary Section 3.1. The within-school contact structure, determining the specific probability of transmission between two pupils attending school, is detailed in Supplementary Section 3.2. External infection to pupils is detailed in Supplementary Section 3.3.

External transmission to pupils and pupil-to-pupil transmission occur independently each time step. If a pupil is infected both via external transmission and from another pupil on the same day, the pupil is assigned randomly as internally or externally infected. Pupils infected via both routes occur only rarely in simulations, accounting for less than 0.03% of infections in the simulations from the main analysis.

The initial prevalence in each school at the start of the simulation depended on that school's LTLA. The initial level of immunity at the start of simulation was set as 6.25%, reflecting the estimated prevalence of COVID-19 antibodies in September 2020 nationally[4].

## 3.1 Pupil-to-pupil transmission

Within the model, the expected number of secondary infections from an infected pupil attending school is influenced by a number of factors, which we expand upon in turn below.

As in our previous study[5], an infected pupil's relative probability of transmission to other pupils on each day since infection was set according to a previously derived infectivity profile for COVID-19[6], based on data from known source-recipient pairs[7] (under the assumption that the generation time and incubation period are independent). Specifically, we assumed that the relative probability of transmission since the

day of infection is given by a gamma distribution ($\Gamma_I(d)$) with shape 5.62 and scale 0.98[6], with an assumed incubation period distribution (gamma distributed with shape 5.807 and scale 0.948[8]). After 14 days, infected individuals recover with immunity that persists over the course of the simulation. We modelled infections from the 24th August 2020 (a week before schools reopened) until 23rd May 2021. We assumed that pupils did not attend school, and therefore did not transmit infection within school, from the 24th-28th August 2020, from 26th-30th October 2020, and from 21st December 2020 - 5th March 2021, corresponding to periods when schools were either on holiday or not fully reopen due to lockdown measures. Additionally, pupils also did not attend school for two weeks out of the three from 29th March 2021 - 16th April 2021, with the specific weeks not attending school dependent on that school's LTLA.

The initial level of transmission within a particular school $s$, $K_s$ was drawn from a lognormal distribution: $K_s \sim \text{LogNormal}(log(K) - \sigma_K^2/2, \sigma_K^2)$, with the parameters $K$ and $\sigma_K$ determined via the fitting procedure, with $K$ representing the mean value of $K_s$ across schools. $K_s$ is a composite term that captures both the contact rate of individual $i$ and the transmission probability across those contacts, which defined the expected number of secondary cases from an infected symptomatic pupil in a particular school, assuming a fully susceptible school population, that pupils attend school each day of their infection, and that the impact of depletion of susceptibles is negligible.

In reality, many secondary school aged children remain asymptomatic throughout infection[9], with asymptomatic individuals likely to be less infectious than symptomatic individuals[10,11]. Hence, we explicitly include asymptomatic transmission in our model, assuming that asymptomatic individuals are less infectious than symptomatic pupils by a factor $a, 0 \leq a \leq 1$ i.e. if a symptomatic pupil is expected to infect $K_s$ other pupils over the course of their infection, an asymptomatic pupil is expected to infect $a \times K_s$ other pupils. We define the function $A_i$ to be such that $A_i = 1$ if a pupil will develop symptoms, or $A_i = a$ otherwise.

The level of pupil-to-pupil transmission on day $d$ is also impacted by the proportion of cases within a school's LTLA that are of the B.1.1.7 (Alpha) variant. This proportion is obtained by fitting a sigmoidal curve to the proportion of PCR tests within an LTLA that return an S-gene negative result out of those that return an S-gene status, (Supplementary Figure 3), a reliable indicator of the B.1.1.7 variant. This proportion scales the level of within-school transmission. We let $V_{LTLA}(d)$ denote the scaling factor increase in within-school transmission resulting from the proportion of the B.1.1.7 variant present in an LTLA on day $d$, with $V_{LTLA}(d) = v, 1 \leq v \leq 2$ when all new cases are of the B.1.1.7 variant, and with $V_{LTLA}(d) = kv$ when a proportion $k$ of new cases are of the B.1.1.7 variant. Our range for the proportional increase in transmissibility of the B.1.1.7 variant (compared to wildtype variants previously in circulation) was chosen to reflect contemporary estimates of the increased transmissibility of the B.1.1.7 variant[12–14].

We also introduce $F(d)$, a function scaling pupil-to-pupil transmission for all schools by a constant factor $f, 0.5 \leq f \leq 3$ from 2nd November 2020 onwards, i.e. after the first half-term of schools reopening. The purpose of this parameter is to account for the impact of reduced adherence to within-school control measures as the school term progresses, after initially very high adherence to such measures. We determine a posterior distribution for $f$ via the fitting procedure. Without the introduction of this parameter, an increase in transmissibility was attributed to the Alpha variant that we deemed as implausible based on estimates from other studies[12–14]. Because of these correspondence issues, we introduced $f$ to obtain a reasonable fit to Pillar 2 testing data through time, whilst still obtaining increases in transmissibility of the B.1.1.7 variant consistent with contemporary estimates. Our prior range for $f$ extends to scenarios that account for a decrease in transmissibility from 2nd November 2020, to assure that the increase in transmissibility found was not an artefact of the choice of prior. All but one value of $f$ from the posterior sample were above 1, with a 95% credible interval of 1.09-2.18.

For a pupil $i$ infected on day $d_0$, attending a school $s$ situated in a particular LTLA on day $d$, the infectiousness of individual $i$ on day $d$ is given by:

$$\lambda_i(d) = \begin{cases} A_i \times F(d) \times V_{LTLA}(d) \times K_s \times \Gamma_I(d - d_0), \text{ if } i \text{ attends school} \\ 0, \text{ otherwise} \end{cases} \quad (1)$$

The interpretation of $\lambda_i(d)$ is the expected number of secondary infections from individual $i$ on day $d$, assuming all their contacts are susceptible. In total, we fit six model parameters relevant to pupil-to-pupil transmission: $K, \sigma_K, a, f, v$ and the proportion of the infected pupils who develop symptoms over the course of their infection.

(a)

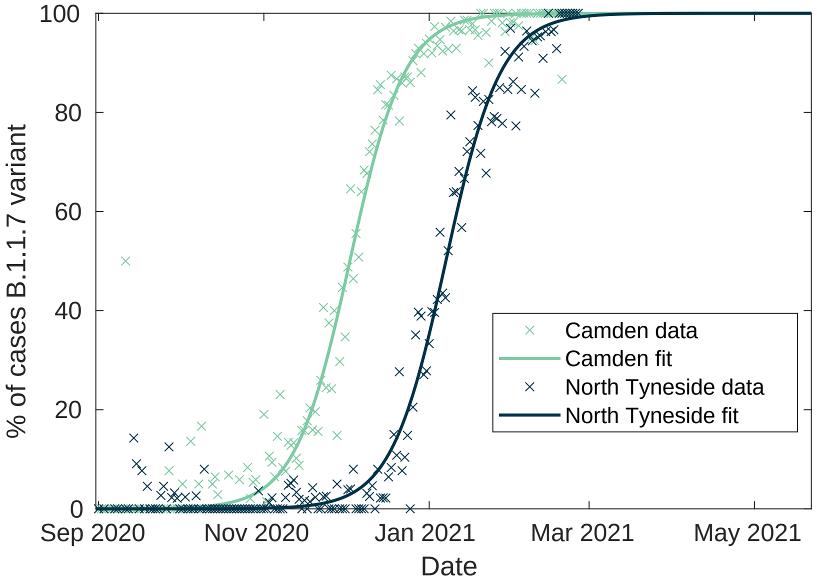


Supplementary Figure 3: **Approximating the relative frequency of B.1.1.7 (Alpha) variant cases.** The percentage of PCR tests from an LTLA that return an S-gene negative result out of those that return an S-gene status (cross markers) increases approximately sigmoidally through time. As an S-gene negative result is a reliable genomic marker of the B.1.1.7 variant, we approximate the percentage of cases of the B.1.1.7 variant through time by fitting a sigmoidal curve to this data (solid lines). We plot these data and the fitted sigmoidal curves for two exemplar LTLAs.

### 3.2 School contact structure

In our previous study, to understand the impact of control measures targeted at year groups [5], we described a situation where secondary schools implemented a bubbling policy at the level of year groups and assumed that pupils mixed randomly within year groups. However, while the majority of secondary schools report implementing a bubbling policy at the level of year groups [15], schools have often isolated smaller groups of targeted close contacts upon confirmation of a positive case [16], in line with the guidance to secondary schools in England as of June 2021 [17]. To account for these variations in the size of groups being isolated, we extended our model to consider three levels of mixing. Each year group was comprised of exclusive sets of close contacts of equal size, with each school comprised of several year groups. We set pupils to mix with their close contacts at a rate $\alpha_0$, mix with pupils in the rest of their year group at a rate $\alpha_1$, and mix with pupils in the rest of their school at a rate $\alpha_2$. By default, we set $\alpha_0 = 1$ and let $0 \leq \alpha_1, \alpha_2 \leq 1$. The hierarchy of $\alpha$ terms reflects relative mixing rates compared to a baseline mixing rate of close contacts - i.e the probability an individual has contact with a given non-close contact member in the same year group is $\alpha_1$ times their probability of contact with a close contact, while the probability an individual has contact with a pupil in a different year group is $\alpha_2$ times their probability of contact with a close contact. Recalling $\lambda_i(d)$ denotes the infectiousness of individual $i$ on day $d$, and letting $N_x$ denote the number of contacts a pupil has of type $x$, we set the probability of transmission from an infectious individual $i$ to a susceptible individual $j$ to be:

$$\tau(i,j) = \frac{\lambda_i(d)}{N_C + \alpha_1 N_Y + \alpha_2 N_S} \times \begin{cases} 1, \text{ if } i \text{ and } j \text{ are close contacts} \\ \alpha_1, \text{ if } i \text{ and } j \text{ are in the same year but not close contacts} \\ \alpha_2, \text{ if } i \text{ and } j \text{ are in different years} \\ 0, \text{ if } j \text{ does not attend school} \end{cases} \tag{2}$$

$$C \in \{\text{Close contacts}\}, Y \in \{\text{Rest of year}\}, S \in \{\text{Rest of school}\} \tag{3}$$

The probability of transmission to a susceptible individual $j$, $\beta_j(d)$, is given by 1 minus the probability that

$j$ is not infected by any pupil on day $d$, i.e.:

$$\beta_j(d) = 1 - \prod_i (1 - \tau(i,j)) \tag{4}$$

In these equations, choosing $\alpha_1 = \alpha_2 = 1$ corresponds to random mixing across the entire school, while at the other extreme, where $\alpha_1 = \alpha_2 = 0$, pupils mix exclusively with their close contacts. In our baseline scenario, we assumed $\alpha_1 = 0.1$ and $\alpha_2 = 0.01$, reflecting a situation where pupils have a much higher rate of interaction with their close contacts than other members of their year group, and where year group bubbles have been in general very effective (with only a small rate of interaction between years to account for the impact of indirect infection via teachers and siblings). To understand the sensitivity of our results to these mixing assumptions, in Supplementary Text 8 we considered a situation with more mixing within-years and across years, setting $\alpha_1 = 1$ and $\alpha_2 = 0.1$: qualitatively similar results were obtained.

### 3.3 External infection

Within the model, the probability of external infection of pupils depended on each school's LTLA and varied through time. Detailed temporal COVID-19 testing data have been recorded at the LTLA level[1], and the positive testing rate within a population is a function of the prevalence in that population. To account for the delay between contracting infection and displaying symptoms, with a mean time from infection to symptom onset of 5.5 days, we assumed that the rate of external infection of school pupils on day $d$ was proportional to the proportion of all individuals in that school's LTLA testing positive to a PCR test on day $d + 5$. This positive testing rate was then scaled by a factor depending on whether a school was situated in a rural or urban area, reflecting reduced exposure to community infection of children in rural communities compared to those in urban areas. For all schools, the probability of external infection was scaled by a factor $h$ during school holidays.

All pupils from a school $s$ in a given LTLA who were not isolating had a probability of external infection each day $d$, proportional to the proportion of the LTLA's population who tested PCR positive on day $d$ (which we denote $X^{LTLA}_{PCRtot}(d)$), obtained from Pillar 2 data. For each school $s$, $X^{LTLA}_{PCRtot}(d)$ is scaled by a factor $\epsilon, 0 < \epsilon \leq 4$.

To reflect reduced exposure to community infection of children in rural communities compared to those in urban areas, the probability of external infection for pupils in rural schools is scaled down by a factor $r, 0 < r \leq 1$. We introduce the function $\rho_S$, which equals 1 for urban schools and $r$ for rural schools. The probability of external infection for pupils is scaled up during school holidays and school closures by a factor $h, 1 \leq h \leq 3$, because during such periods pupils will spend more time with non-school contacts, hence increasing their risk of community infection. We introduce the function $H(d)$ which equals 1 during term time and $h$ during school holidays or closures. For a susceptible pupil $j$, who attends school $s$ situated in a particular LTLA, the probability that they are infected via community infection is given by $E_s(d)$:

$$E_s(d) = \rho_S \times H(d) \times \epsilon \times X^{LTLA}_{PCRtot}(d) \tag{5}$$

Pupil $j$'s total probability of infection on day $d$ is given by $1 - ((1 - \beta_j(d)) \times (1 - E_s(d)))$, with $\beta_j(d)$ as defined in Equation (4).

In total, we fit three model parameters relevant to external infection via community transmission: $\epsilon, r$ and $h$.

## Supplementary Text 4 Testing in detail

### 4.1 School testing policies

Throughout the simulations, all symptomatic infected pupils underwent a PCR test upon symptom onset. The period to symptom onset was drawn from a Gamma distribution with shape 5.807 and scale 0.948[8]). Pupils self-isolated until they received a test result. We assumed that pupils received a result two days after taking a test (though in reality there will be some variation in the length of time between taking a test and receiving a result). Those receiving a negative result returned to school the day after receiving this

notification, while those testing positive entered isolation for a period including the day that symptom onset began and the next ten full days[18]. Those testing positive are recorded as a confirmed case of COVID-19 over the course of their isolation period. As asymptomatic pupils did not develop symptoms, they were only detectable through LFT testing. Pupils who tested positive using an LFT entered isolation, with the outcome of a confirmatory PCR test then determining whether the pupil remained in isolation (for a period including the day the LFT test was taken and the next ten full days[18]). Twice weekly mass testing in schools began on the 1st March 2021, i.e. the week before schools reopened. In each school, each pupil was assigned two days of the week (Sunday and Wednesday, Monday and Thursday, etc.) in which they were scheduled to undertake an LFT test each week. The probability of those pupils taking a test on that given day was matched to the uptake of LFTs in that school's LTLA. As LFTs have predominantly been taken at home, we assumed that identified infected pupils did not transmit infection on the day they undertook a test.

Upon a pupil testing positive to a PCR test, the close contacts of that pupil did not attend school for ten days counted from the day of last contact[19]. We refer to this as the isolation of close contacts, consistent with the language used to describe school policies in the event of a confirmed case in the UK[17], though this could also be described as the quarantine of close contacts. Secondary schools isolated the close contacts of infected pupils upon a pupil testing positive to a PCR test (either through self-seeking or as a confirmatory test to a positive LFT) for ten days following the day of last contact[19]. Within the model, the size of close contact groups for each school remained constant over each term, informed by reported absences data. To reflect that schools have in general isolated smaller groups of pupils from March 2021 onwards, the size of close contact groups therefore differed between September to December 2020 and March to May 2021 (Supplementary Figure 2b).

### 4.2 Test sensitivity and specificity

We used previously estimated LFT and PCR test sensitivity profiles for symptomatic individuals[20]. For asymptomatic individuals, we assumed that the probability of testing positive was equal to that of symptomatic individuals until peak positive test probability, but then decayed more rapidly, as in our previous study[5]. The specificity of PCR tests was assumed to be 1, in line with data indicating that false PCR positives are very rare[21]. The specificity of LFTs through mass testing in schools in England has been very high, with a specificity of at least 99.9% reported[22,23] In line with previously obtained estimates of LFT specificity in this setting, we assume the specificity of LFTs to be 99.97%[23].

### 4.3 Twice weekly mass testing

Within the model, twice weekly mass testing of secondary school pupils is implemented from the 1st March 2021 until the end of the simulation, in line with the introduction of mass testing prior to secondary schools reopening on the 8th March 2021. Each pupil was assigned two days of the week they are scheduled to take their tests - taking their first test on Sunday, Monday, Tuesday, or Wednesday, and taking their second test three days after (Wednesday, Thursday, Friday, or Saturday, respectively). The probability of a pupil scheduled to take a test $p$ was adjusted to match daily LFT participation in secondary school aged pupils within that school's LTLA.

Daily LFT participation per LTLA was taken as the proportion of 10-19 year olds in that LTLA who recorded either a positive or negative LFT on that day. We used the broader age range of 10-19 year olds here due to the negative Pillar 2 data only being available in five year age bands. This is likely an underestimate, for two reasons. Firstly, a considerable proportion of negative home tests remain unrecorded. To account for this, we determine the level of underreporting, $u, 1 \leq u \leq 4$, via the fitting procedure. The number of negative LFTs per LTLA is scaled up by the factor $u$ from 14th March 2021, i.e. after tests shifted from being at school to being taken at home. We assume that $u$ remains constant over time and is constant across LTLAs, though in reality $u$ may wane over time and vary by LTLA. Secondly, the age range considered (10-19 year olds) includes some ages not attending secondary school (who are not expected to take LFT tests), and consequently testing uptake is likely concentrated in a smaller age range. While this is not accounted for explicitly, the scaling up via underreporting counteracts this underestimation. LFT participation levels obtained from data and modelled participation after accounting for underreporting is shown in Supplementary Figure 4a.

Estimates of LFT participation levels per LTLA over the term are taken as the mean daily LFT participation from 8th March 2021 until 23rd May 2021, divided by 2/7 - as 2/7ths of pupils taking a test every day would correspond to full uptake of twice weekly mass testing (assuming that the day of first tests and day

of second tests are distinct and random). There is considerable heterogeneity in LFT participation between LTLAs, shown in Supplementary Figure 4b.

(a)

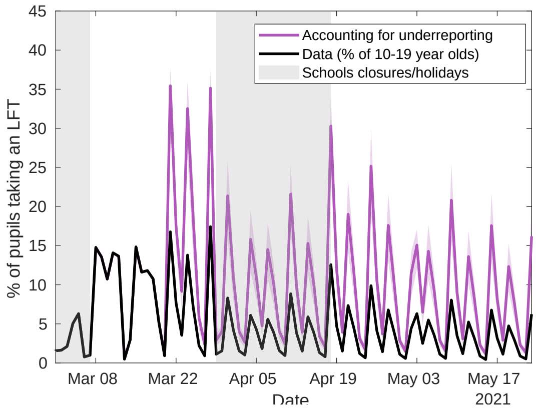


(b)

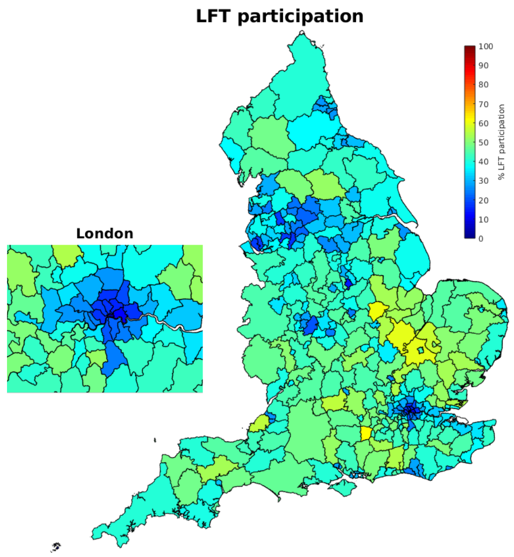


Supplementary Figure 4: **Modelling LFT participation.** (a) We plot time-series of the percentage of 10-19 year olds in England who report either a positive or negative LFT result each day (black line), and of the modelled percentage of pupils who take an LFT each day after accounting for underreporting of home tests from 21st March 2021 (purple line). Model results are from 100 simulations of 2979 secondary schools, each with a distinct parameter set sample from the posterior distribution, with the solid purple line corresponding to the mean estimate and the shaded purple interval representing the 95% prediction interval. (b) LTLAs shaded according to modelled percentage participation level of LFTs from 8th March-23rd May 2021, taken as the mean daily LFT participation within that LTLA from 8th March 2021 until 23rd May 2021, divided by 2/7.

# Supplementary Text 5 Model fitting

## 5.1 The inference scheme

The model is fitted using an Approximate Bayesian Computation Sequential Monte Carlo (ABC-SMC) approach[24,25]. For the first generation, we sampled particles from the prior distribution (Supplementary Table 1), until we obtained 200 particles returning finite log-likelihoods. The covariance matrix of these particles informed the covariance matrix for the perturbation kernel, used to generate candidate particles from the particles retained in the previous generation. Our perturbation kernel was 0.68 times the weighted empirical variance of the particles retained in the previous generation. After the first generation, we generated 200 accepted parameter sets for each generation that returned log-likelihoods greater than the median log-likelihood of particles in the previous generation.

To navigate parameter space more efficiently, we first performed the fitting for a representative sample of 100 schools for ten generations. We then ran the model fitting scheme for a representative sample of 600 schools ($\approx 20\%$ of the schools) for a further eight generations. Representative samples of schools were chosen such that the distribution of school sizes, the distributions of close contact sizes, and the mean community testing rate per pupil matched closely the whole set of schools (Supplementary Figure 5).

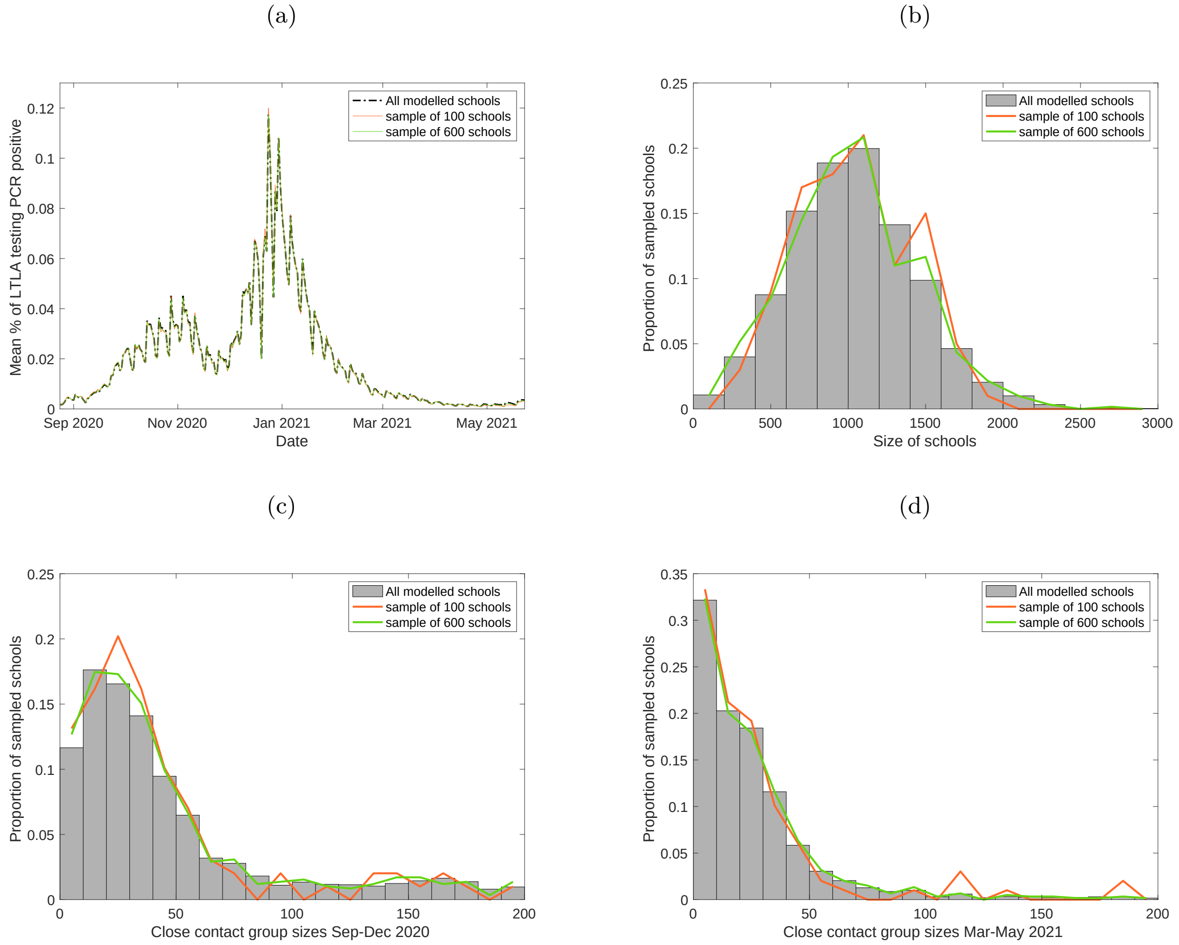


Supplementary Figure 5: **Representative sample of schools for model fitting.** The above plots compare the sample of 100 schools used for the first 11 generations of model fitting (orange) and the sample of 600 schools used for the subsequent 8 generations of model fitting (green) against the four characteristics of the whole set of modelled schools (black dot-dashed in (a); grey in (b-d)) they were matched against: (a) the mean community testing rate per pupil, calculating by averaging the percentage of an LTLA who tested positive to a Pillar 2 PCR test each day over all modelled pupils, (b) the distribution of modelled school sizes, (c) the distribution of close contact group sizes from 1st September 2020 to 18th December 2020 and (d) the distribution of close contact group sizes from 8th March 2021 to 23rd May 2021.

When running the fitting scheme using the representative sample set of 600 schools, to evaluate the log-

likelihood for a candidate particle we employed a two-step process to improve algorithmic efficiency. Firstly, we initially evaluated the log-likelihood using 100 schools. If the log-likelihood was below the median log-likelihood obtained from the tenth generation, the parameter set was discarded. If the log-likelihood was above the median log-likelihood obtained from the tenth generation, we simulated epidemics in the remaining 500 schools and recalculated the log-likelihood.

### 5.2 The log-likelihood function

We fitted the model to Pillar 2 testing data and peak confirmed cases as recorded in the schools data (as described in Supplementary Text S1). We could define the log-likelihood of the model parameters given the data for each of the data streams. We based the time-series components of the log-likelihood function on a binomial likelihood for each week. We let $X_T(w)$ denote the observed number of positive tests of type $T, T \in \{\mathrm{PCR}, \mathrm{LFT}\}$ in 11-16 year olds on week $w$, $Y_T(w)$ denote the predicted proportion of pupils who test positive on week $w$ to a test of type $T$, and we let $N$ denote the population size of 11-16 year olds in England. $L_B(n|N,p)$ denotes the log of the binomial probability function, with $n$ the number of observations out of a total population of size $N$ given a probability of event occurrence $p$. We assumed the components corresponding to the distributions of peak confirmed cases in schools followed a multinomial distribution. We let $S_P(k)$ denote the number of secondary schools with a peak of $k$ confirmed cases over period $P, P \in \{\text{September to December 2020 } (septodec), \text{March to May 2021 } (martomay)\}$, $Y_P(k)$ denote the predicted proportion of schools with a peak of $k$ confirmed cases over period $P$. For September to December 2020, we aggregated schools recording 15 confirmed cases or above into the same bin, while for March to May 2021 we aggregated schools recording 8 confirmed cases or above into the same bin. These aggregation thresholds were chosen such that each bin contained at least 2% of schools recording data for that period. If no simulated schools recorded $k$ confirmed cases, we added 1 to the number of simulated schools recorded in each bin to avoid obtaining log-likelihoods of $-\infty$. In sum, our log-likelihood function was:

$$\begin{aligned} LL(\theta) = &\sum_w L_B(X_{PCR}(w)|N, Y_{PCR}(w)) + \sum_w L_B(X_{LFT}(w)|N, Y_{LFT}(w)) \\ &+ \sum_k [S_{septodec}(k) \times log(Y_{septodec}(k))] + \sum_k [S_{martomay}(k) \times log(Y_{martomay}(k))]. \end{aligned} \quad (6)$$

### 5.3 Inspection of parameter posterior distributions

Supplementary Table 1 describes and explains each model parameter, the prior distribution assumed for each parameter, and the 95% credible interval of the posterior distribution for each parameter.

Supplementary Figure 6 displays density plots of a sample of 200 parameter sets obtained from the posterior distribution, while Supplementary Figure 7 displays pairwise scatter plots of the fitted values of each parameter. $K$ was negatively correlated with all other parameters impacting pupil-to-pupil transmission ($\sigma_K, f, v, a$ and the proportion of symptomatic individuals), moderating the increase in transmission from higher values of $K$. Supplementary Figure 8 demonstrates the improvement in fit obtained in successive generations via the fitting process, plotting log-likelihood values obtained each generation.

Supplementary Table 1: **Explanation of model parameters, assumed prior distributions, and posterior ranges.** All credible interval ranges are specified to two decimal places. We drew within-school transmission for each school $K_s$ from a lognormal distribution: $K_s \sim \text{LogNormal}(log(K) - \sigma_K^2/2, \sigma_K^2)$.

| **Parameter** | **Explanation** | **Prior** | **Posterior (95% credible interval)** |
|---|---|---|---|
| 1. Mean pupil-to-pupil transmission parameter, $K$ | Initial expected secondary infections from a symptomatic pupil in a completely susceptible school, with replacement of infected pupils. | $\text{Unif}(0, 4)$ | 0.90-2.77 |
| 2. $\sigma_K$ parameter of pupil-to-pupil transmission lognormal distribution | Schools likely vary in efficacy of control measures. | $\text{Unif}(0, 1)$ | 0.13-0.70 |
| 3. Scaling factor of increased pupil-to-pupil transmission after October half-term, $f$ | Reducing adherence to within-school control measures as the school term progresses. | $\text{Unif}(0.5, 3)$ | 1.09-2.18 |
| 4. Increase in transmissibility of the B.1.1.7 variant, $v$ | More transmissible variants will increase pupil-to-pupil transmission [12,14,26]. | $\text{Unif}(1, 2)$ | 1.60-1.98 |
| 5. Scaling constant for community infection, $\epsilon$ | Probability of external infection is proportional to community prevalence. | $\text{Unif}(0, 4)$ | 1.02-2.20 |
| 6. Scaling factor of community infection for rural schools, $r$ | Pupils at school in rural areas likely have less exposure to infection in the community. | $\text{Unif}(0, 1)$ | 0.10-0.85 |
| 7. Scaling factor of community infection during school holidays, $h$. | During school holidays pupils spend more time with non-school contacts, increasing risk of community infection. | $\text{Unif}(1, 3)$ | 1.18-2.50 |
| 9. Relative infectiousness of asymptomatic pupils, $a$ | Asymptomatic pupils are likely less infectious than symptomatic pupils [10,11]. | $\text{Unif}(0, 1)$ | 0.14-0.93 |
| 8. Proportion of pupils who are symptomatic | Many secondary-school aged children will be asymptomatic and will not seek PCR tests [9]. | $\text{Unif}(0, 1)$ | 0.35-0.58 |
| 10. Underreporting of negative home LFT tests, $u$ | Pupils may not always accurately report LFTs taken at home. | $\text{Unif}(1, 4)$ | 1.96-3.24 |

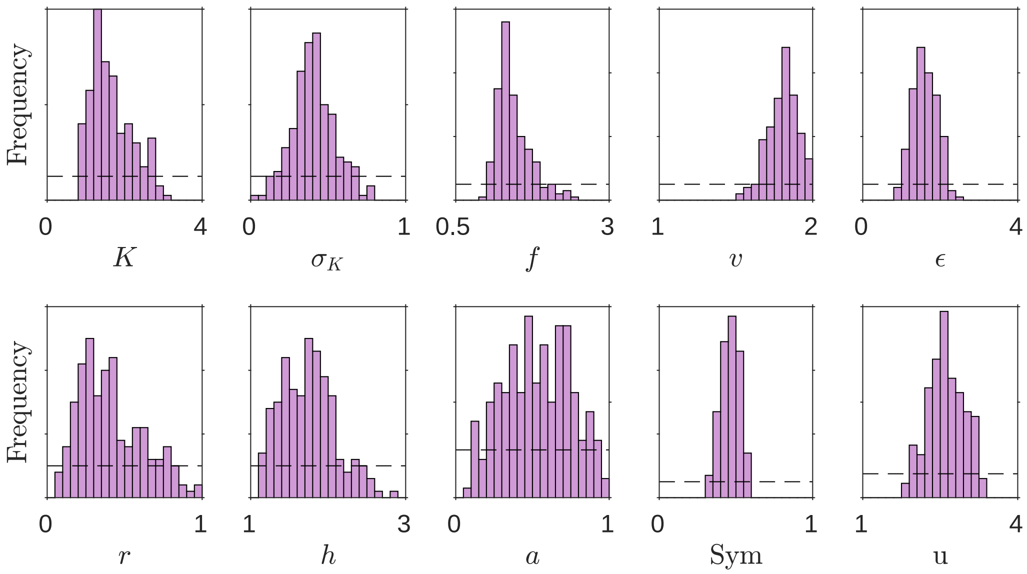


Supplementary Figure 6: **Posterior distribution density plots.** Posterior parameter density plots for each of the parameters fitted in the model via an ABC-SMC approach for the parameters: $K$ and $\sigma_K$, the parameters determining the baseline pupil-to-pupil transmission parameter $K_s \sim \text{LogNormal}(log(K) - \sigma_K^2/2, \sigma_K^2)$; $f$, the scaling factor on pupil-to-pupil transmission after the October 2020 half-term; $v$, the increased transmissibility of the new variant; $\epsilon$, the scaling constant for community infection; $r$, the scaling factor of community infection for rural schools; $h$, the scaling factor of community infection during school holidays; $a$, the relative infectiousness of asymptomatic pupils; $Sym$, the proportion of pupils who are symptomatic; and $u$, the underreporting of negative home LFTs. We constructed the density plots from 200 parameter sets sampled from the posterior distribution. Dashed lines indicate the uniform prior distribution of the parameter. We observe the density of most parameter to be approximately normally distributed.

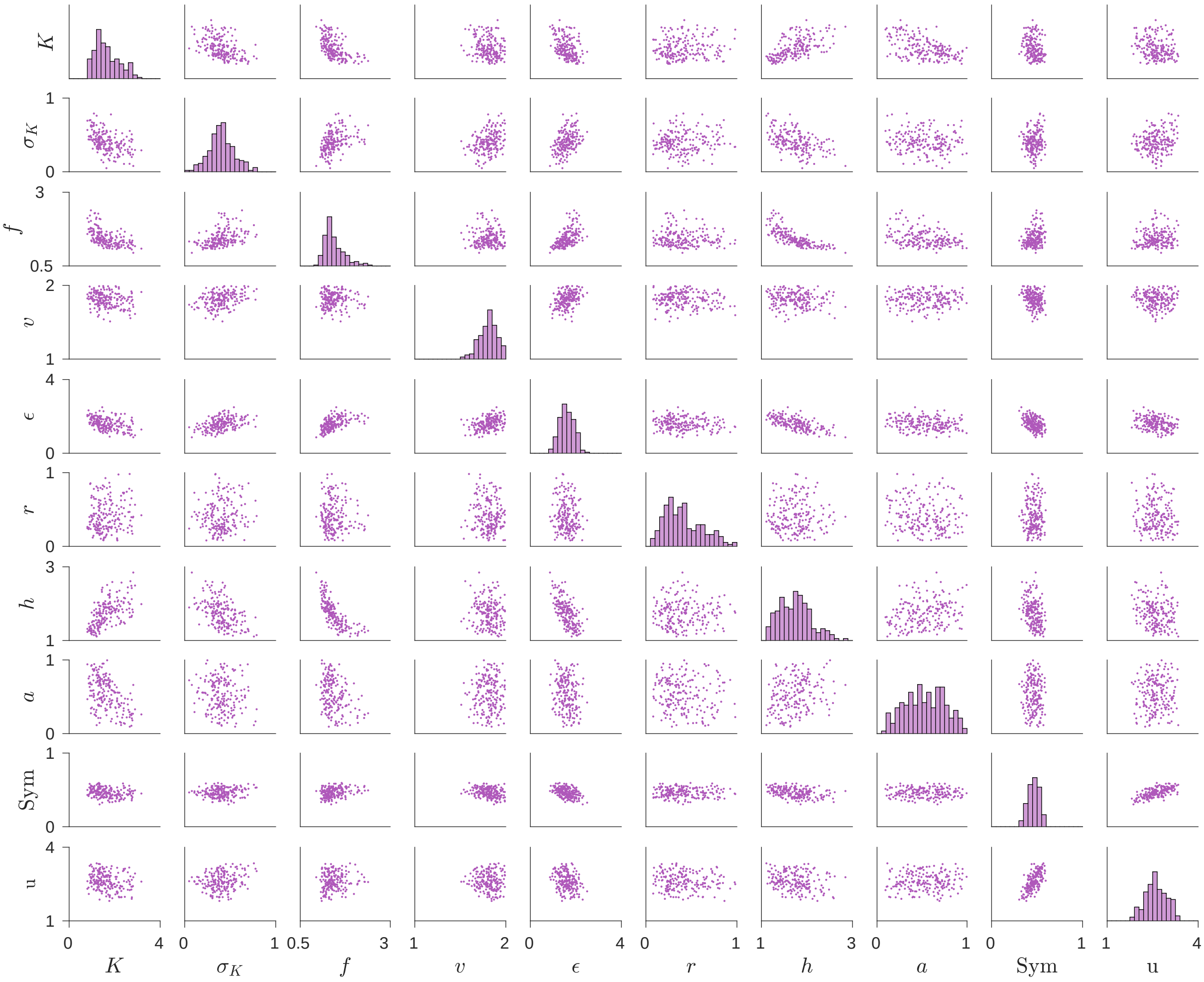


Supplementary Figure 7: **Pairwise scatter plots of the posterior distribution.** Pairwise scatter plots of 200 parameter sets sampled from the posterior distribution, showing the correlation between the parameters: $K$ and $\sigma_K$, the parameters determining the baseline pupil-to-pupil transmission parameter $K_s \sim \text{LogNormal}(log(K) - \sigma_K^2/2, \sigma_K^2)$; $f$, the scaling factor on pupil-to-pupil transmission after the October 2020 half-term; $v$, the increased transmissibility of the new variant; $\epsilon$, the scaling constant for community infection; $r$, the scaling factor of community infection for rural schools; $h$, the scaling factor of community infection during school holidays; $a$, the relative infectiousness of asymptomatic pupils; $Sym$, the proportion of pupils who are symptomatic; and $u$, the underreporting of negative home LFTs. Diagonal entries show posterior parameter density plots of each parameter.

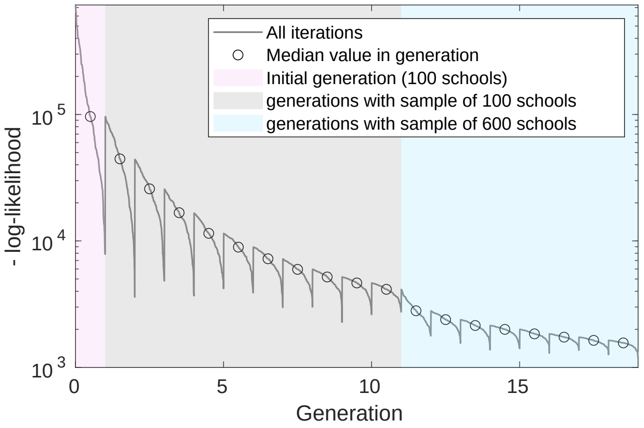


Supplementary Figure 8: **Model fitting by generation.** We display negative log-likelihood values obtained during the model fitting process for the accepted parameters of each generation. The dark grey line displays the negative log-likelihood values of all accepted parameter sets, plotted in descending order for each generation. Circles indicate the median log-likelihood of each generation, which is the value used as the threshold of acceptance for the subsequent generation. The initial generation is shaded in pink, where parameter sets are drawn randomly from the prior distribution until, upon simulation of epidemics in a sample of 100 schools, finite log-likelihoods are returned. The initial generation is followed by 10 generations, shaded in grey, where the model also simulates epidemics in a sample of 100 schools. This is followed by 8 generations, where the model simulates epidemics in a sample of 600 schools.

### 5.4 Robustness of model fitting

To explore whether the parameters estimated were robustly inferred by our model fitting approach, we fitted our model to five synthetic data sets. Each synthetic data set was generated by randomly sampling parameters from their respective prior distribution. We ran 100 simulations for each parameter set. We took $X_{PCR}$, $X_{LFT}$, $S_{septodec}$, and $S_{martomay}$ as their average values from the 100 simulations. After fitting to each synthetic data set, we compared the posterior distribution of parameters to the 'true' parameter value used to generate the synthetic data. All posterior distributions contained the parameter values used to generate the synthetic data (Supplementary Figure 9).

The data were consistently informative over the priors for the parameters $K$ - the baseline pupil-to-pupil transmission parameter, $v$ - the increased transmissibility of the Alpha variant, $\epsilon$ - the scaling constant for community infection, $Sym$ - the proportion of pupils who will become symptomatic after infection, and $u$ - the level of underreporting of home LFTs. For most synthetic sets, the data were also informative for the parameters $\sigma_K$ - determining variability between $K_s$ values drawn for each school, $f$ - the scaling factor impacting transmission after the October half-term break, and $h$ - the scaling factor increasing community infection during school holidays. The range of posterior values remained wide in most instances for the parameters $r$ - determining the scaling factor for rural schools, and $a$ - determining the relative infectiousness of asymptomatic individuals. The correlations observed between parameters fitted to the real data were qualitatively similar to those observed in the synthetic data sets (Supplementary Figures 10 to 14).

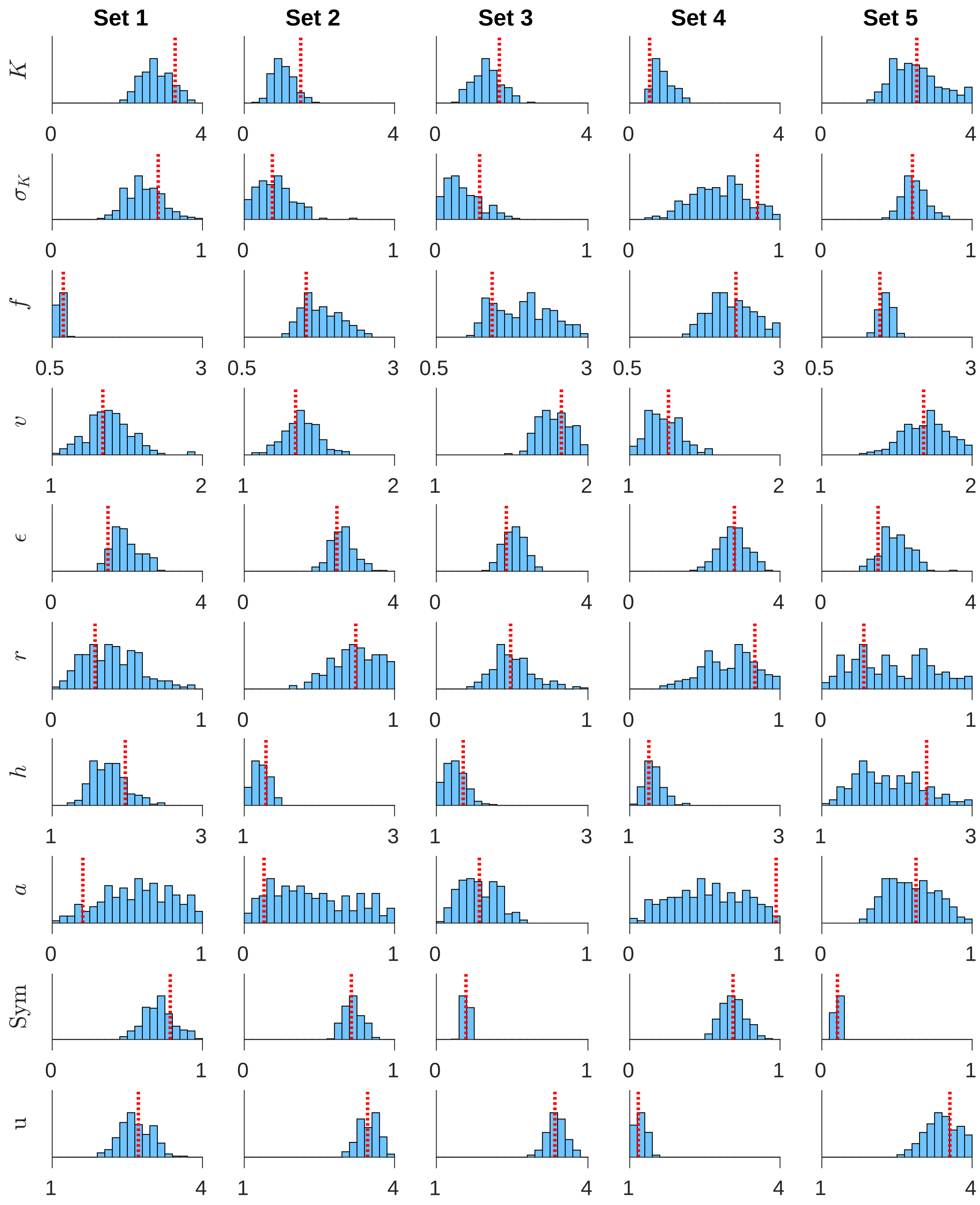


Supplementary Figure 9: **Posterior distribution density plots for five synthetic data sets.** Posterior parameter density plots for each of the parameters fitted in the model via an ABC-SMC approach for five different synthetic data sets. Each column corresponds to one of the five synthetic data sets. The rows show the density plots for parameters (from top to bottom): $K$ and $\sigma_K$, the parameters determining the baseline pupil-to-pupil transmission parameter $K_s \sim \text{LogNormal}(log(K) - \sigma_K^2/2, \sigma_K^2)$; $f$, the scaling factor on pupil-to-pupil transmission after the October half-term; $v$, the increased transmissibility of the Alpha variant; $\epsilon$, the scaling constant for community infection; $r$, the scaling factor of community infection for rural schools; $h$, the scaling factor of community infection during school holidays; $a$, the relative infectiousness of asymptomatic pupils; $Sym$, the proportion of pupils who are symptomatic; and $u$, the underreporting of negative home LFTs. We constructed the density plots from 200 parameter sets sampled from the posterior distribution. Red dotted lines indicate the true parameter value used to generate the synthetic data set.

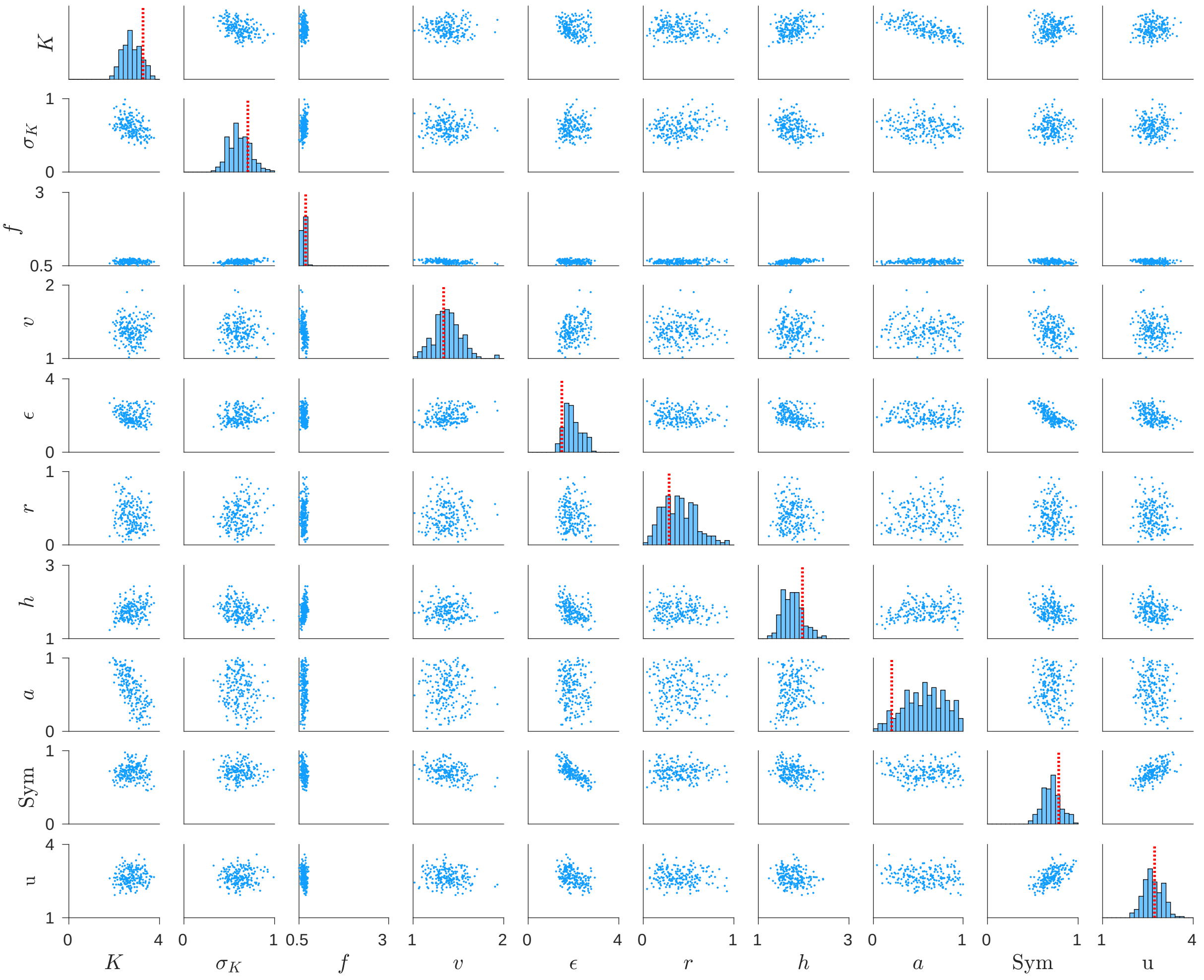


Supplementary Figure 10: **Pairwise scatter plots of the posterior distribution for Synthetic data set 1.** Pairwise scatter plots of 200 parameter sets sampled from the posterior distribution, showing the correlation between the parameters: $K$ and $\sigma_K$, the parameters determining the baseline pupil-to-pupil transmission parameter $K_s \sim \text{LogNormal}(log(K) - \sigma_K^2/2, \sigma_K^2)$; $f$, the scaling factor on pupil-to-pupil transmission after the October 2020 half-term; $v$, the increased transmissibility of the Alpha variant; $\epsilon$, the scaling constant for community infection; $r$, the scaling factor of community infection for rural schools; $h$, the scaling factor of community infection during school holidays; $a$, the relative infectiousness of asymptomatic pupils; $Sym$, the proportion of pupils who are symptomatic; and $u$, the underreporting of negative home LFTs. Diagonal entries show posterior parameter density plots of each parameter. Black dotted lines indicate the true parameter value used to generate the synthetic data set.

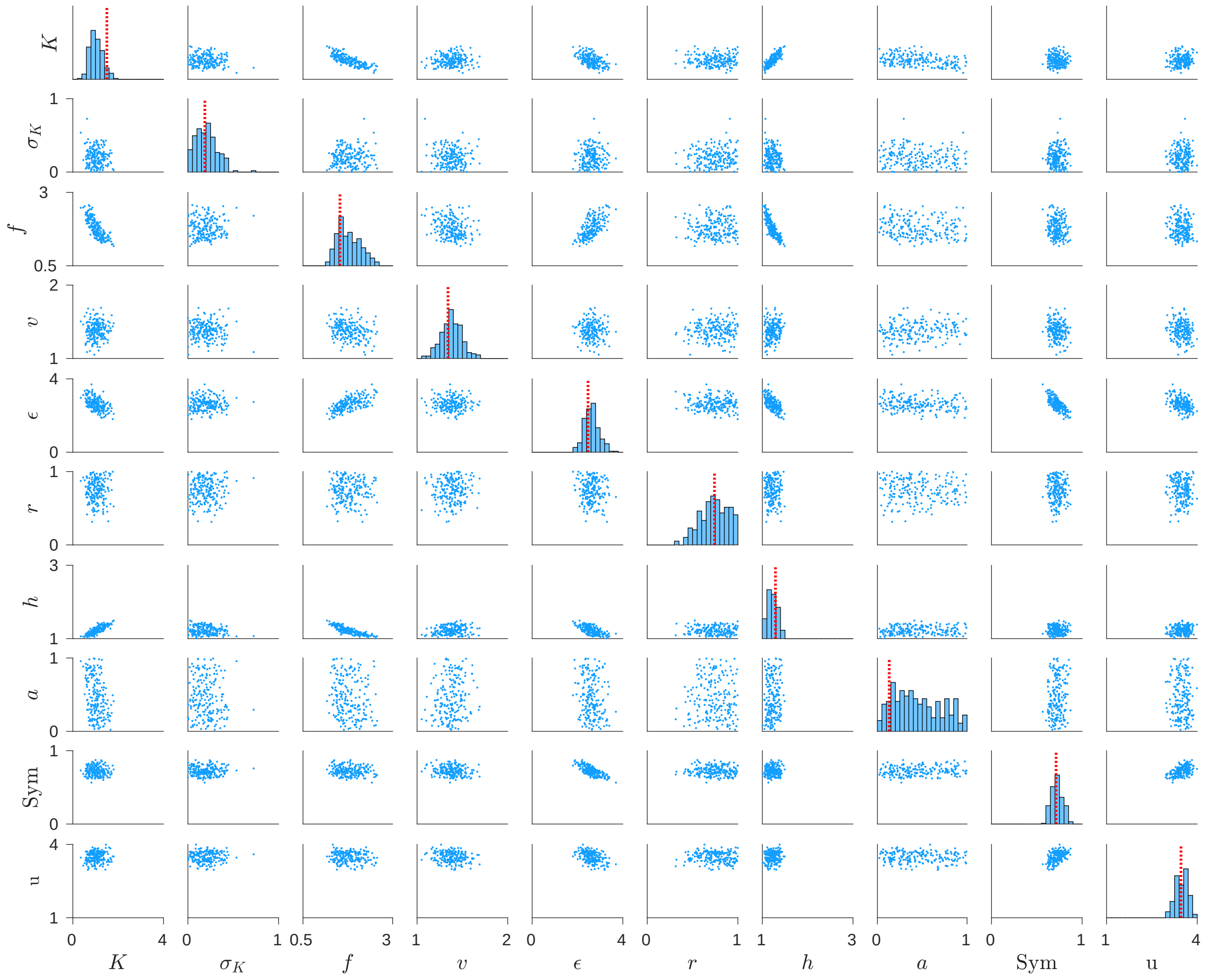


Supplementary Figure 11: **Pairwise scatter plots of the posterior distribution for Synthetic data set 2**. Pairwise scatter plots of 200 parameter sets sampled from the posterior distribution, showing the correlation between the parameters: $K$ and $\sigma_K$, the parameters determining the baseline pupil-to-pupil transmission parameter $K_s \sim \text{LogNormal}(log(K) - \sigma_K^2/2, \sigma_K^2)$; $f$, the scaling factor on pupil-to-pupil transmission after the October 2020 half-term; $v$, the increased transmissibility of the Alpha variant; $\epsilon$, the scaling constant for community infection; $r$, the scaling factor of community infection for rural schools; $h$, the scaling factor of community infection during school holidays; $a$, the relative infectiousness of asymptomatic pupils; $Sym$, the proportion of pupils who are symptomatic; and $u$, the underreporting of negative home LFTs. Diagonal entries show posterior parameter density plots of each parameter. Black dotted lines indicate the true parameter value used to generate the synthetic data set.

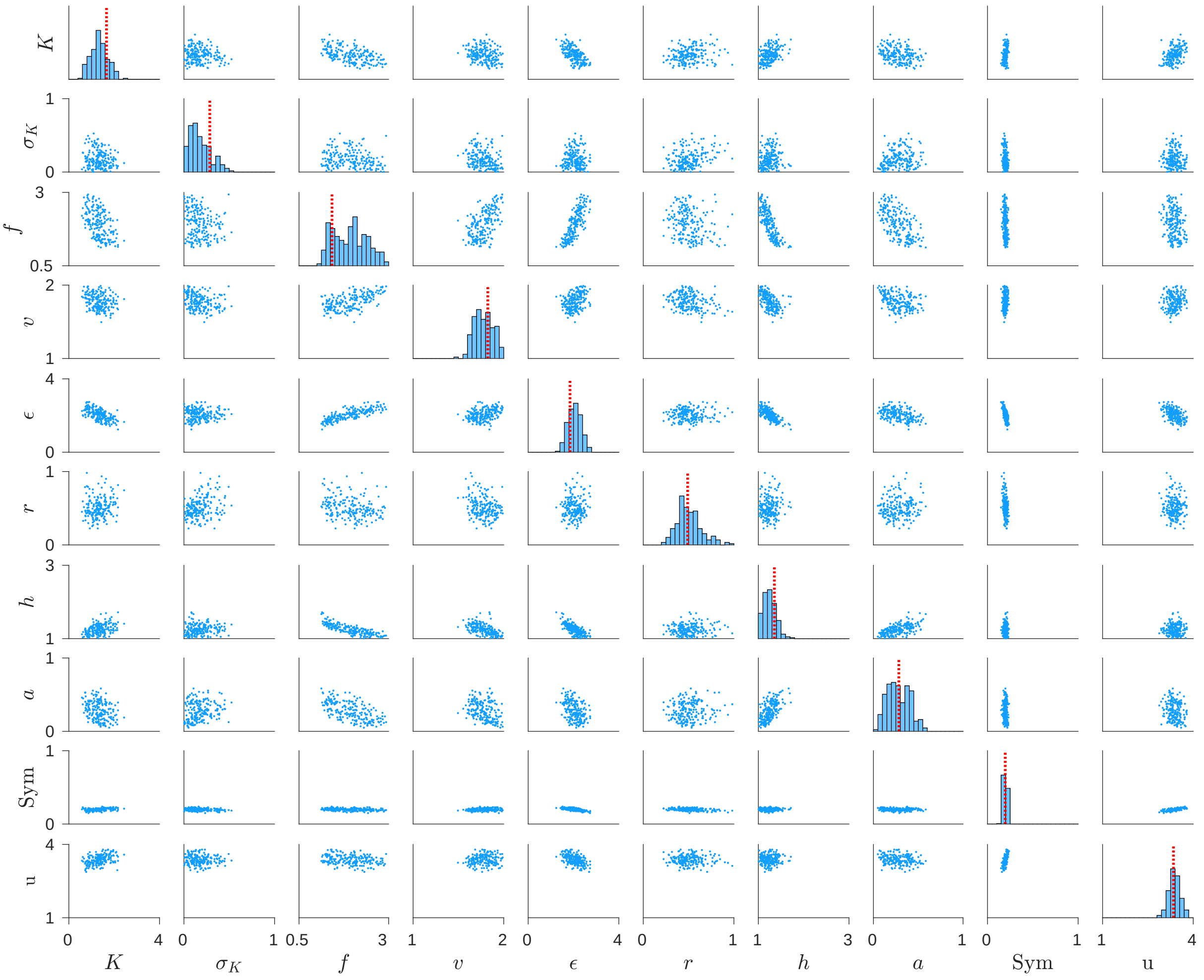


Supplementary Figure 12: **Pairwise scatter plots of the posterior distribution for Synthetic data set 3**. Pairwise scatter plots of 200 parameter sets sampled from the posterior distribution, showing the correlation between the parameters: $K$ and $\sigma_K$, the parameters determining the baseline pupil-to-pupil transmission parameter $K_s \sim \text{LogNormal}(log(K) - \sigma_K^2/2, \sigma_K^2)$; $f$, the scaling factor on pupil-to-pupil transmission after the October 2020 half-term; $v$, the increased transmissibility of the Alpha variant; $\epsilon$, the scaling constant for community infection; $r$, the scaling factor of community infection for rural schools; $h$, the scaling factor of community infection during school holidays; $a$, the relative infectiousness of asymptomatic pupils; $Sym$, the proportion of pupils who are symptomatic; and $u$, the underreporting of negative home LFTs. Diagonal entries show posterior parameter density plots of each parameter. Black dotted lines indicate the true parameter value used to generate the synthetic data set.

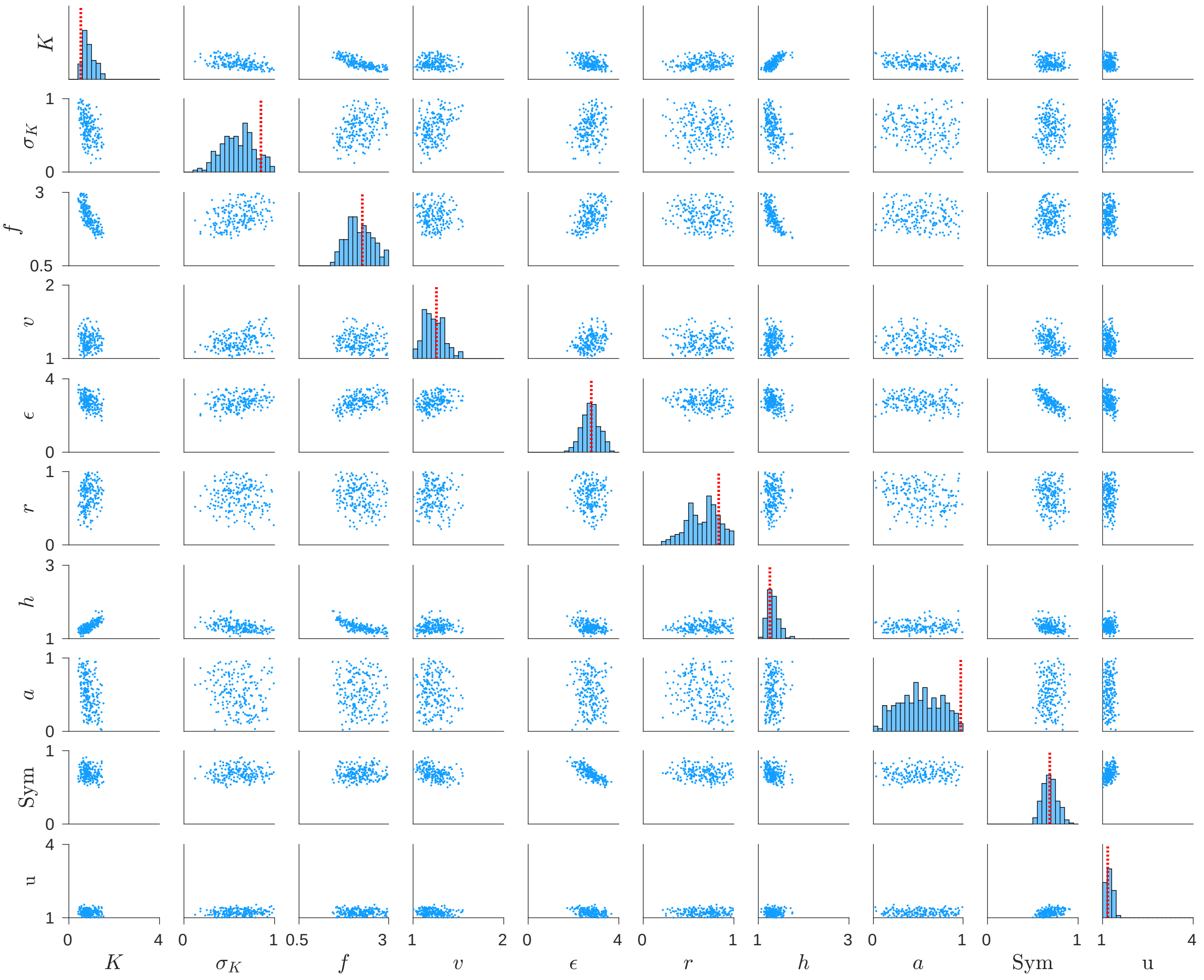


Supplementary Figure 13: **Pairwise scatter plots of the posterior distribution for Synthetic data set 4**. Pairwise scatter plots of 200 parameter sets sampled from the posterior distribution, showing the correlation between the parameters: $K$ and $\sigma_K$, the parameters determining the baseline pupil-to-pupil transmission parameter $K_s \sim \text{LogNormal}(log(K) - \sigma_K^2/2, \sigma_K^2)$; $f$, the scaling factor on pupil-to-pupil transmission after the October 2020 half-term; $v$, the increased transmissibility of the Alpha variant; $\epsilon$, the scaling constant for community infection; $r$, the scaling factor of community infection for rural schools; $h$, the scaling factor of community infection during school holidays; $a$, the relative infectiousness of asymptomatic pupils; $Sym$, the proportion of pupils who are symptomatic; and $u$, the underreporting of negative home LFTs. Diagonal entries show posterior parameter density plots of each parameter. Black dotted lines indicate the true parameter value used to generate the synthetic data set.

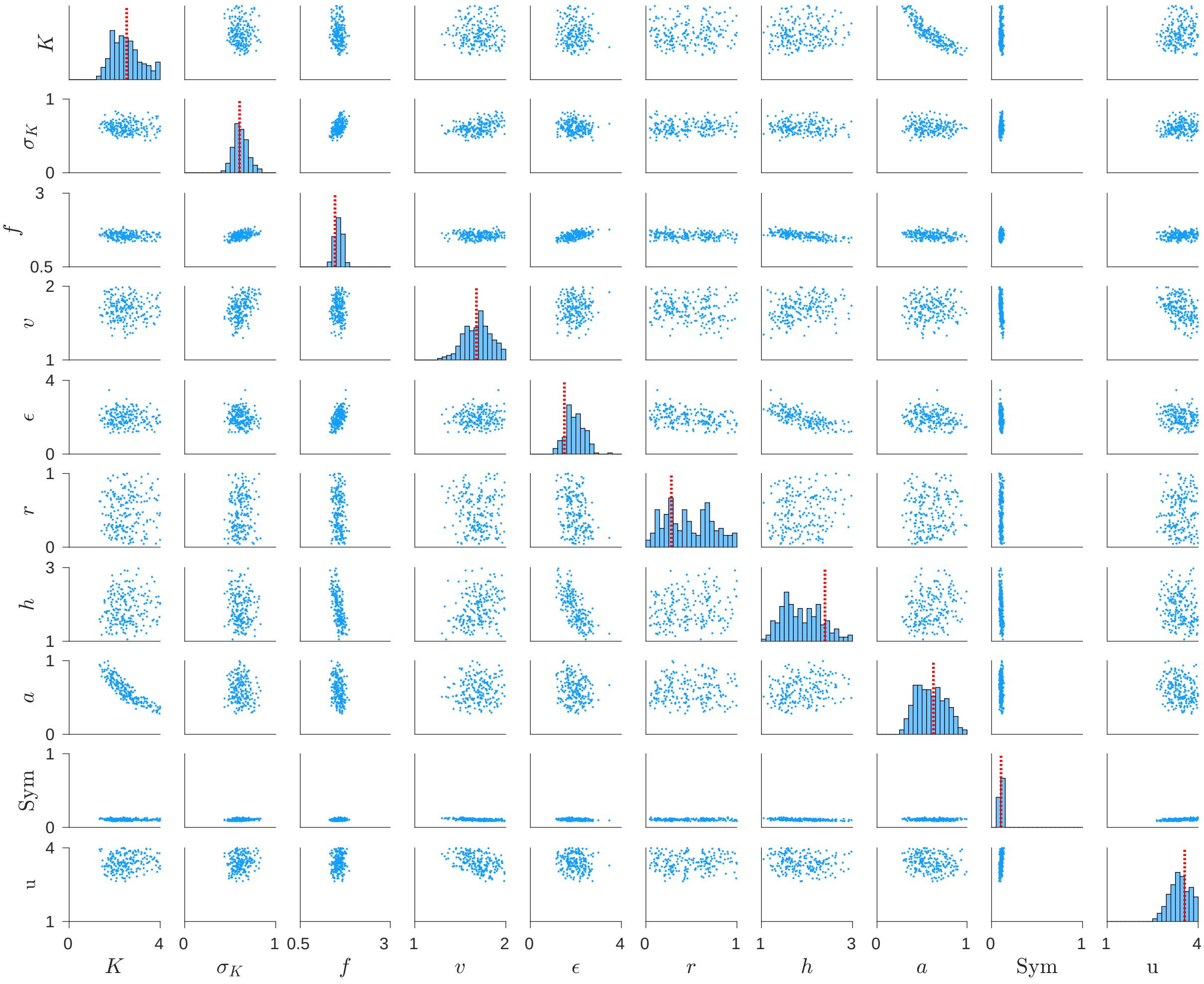


Supplementary Figure 14: **Pairwise scatter plots of the posterior distribution for Synthetic data set 5**. Pairwise scatter plots of 200 parameter sets sampled from the posterior distribution, showing the correlation between the parameters: $K$ and $\sigma_K$, the parameters determining the baseline pupil-to-pupil transmission parameter $K_s \sim \text{LogNormal}(log(K) - \sigma_K^2/2, \sigma_K^2)$; $f$, the scaling factor on pupil-to-pupil transmission after the October 2020 half-term; $v$, the increased transmissibility of the Alpha variant; $\epsilon$, the scaling constant for community infection; $r$, the scaling factor of community infection for rural schools; $h$, the scaling factor of community infection during school holidays; $a$, the relative infectiousness of asymptomatic pupils; $Sym$, the proportion of pupils who are symptomatic; and $u$, the underreporting of negative home LFTs. Diagonal entries show posterior parameter density plots of each parameter. Black dotted lines indicate the true parameter value used to generate the synthetic data set.

# Supplementary Text 6 Comparing the model against testing data by region and testing data by LTLA

We compared the fitted model to Pillar 2 testing data regionally and by LTLA. The model matched PCR testing data closely at a regional level (Supplementary Figure 15), although the model underestimated the heterogeneity in PCR and LFT testing data between LTLAs (Supplementary Figure 16).

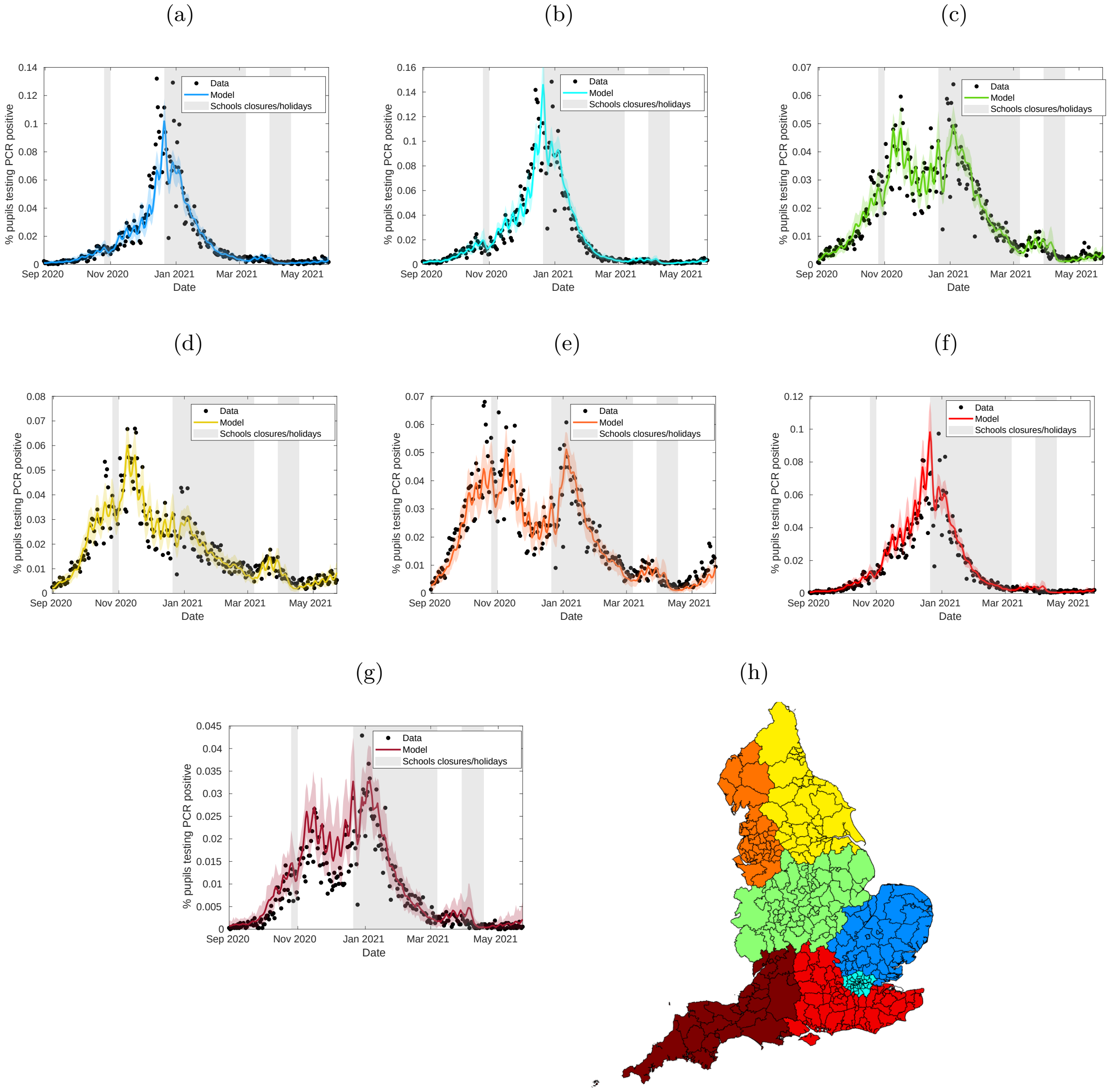


Supplementary Figure 15: **Comparing the fitted model against PCR testing data by region.** The above plots compare the percentage of 11-16 year olds who test PCR positive regionally (excluding confirmatory PCR tests) each day from 1st September 2020 to 23rd May 2021 against the modelled percentage of pupils testing PCR positive through time, for (a) East of England, (b) Greater London, (c), the Midlands, (d) the North-East, (e), the North-West, (f) the South-East, and (g) the South-West, with (h) showing the LTLAs that aggregated together formed each region, with colours corresponding to the time-series plots. We obtained these results from 100 simulations of 2979 secondary schools, each with a distinct parameter set sample from the posterior distribution. In all panels, solid lines correspond to mean temporal profiles and shaded ribbons represent 95% prediction intervals.

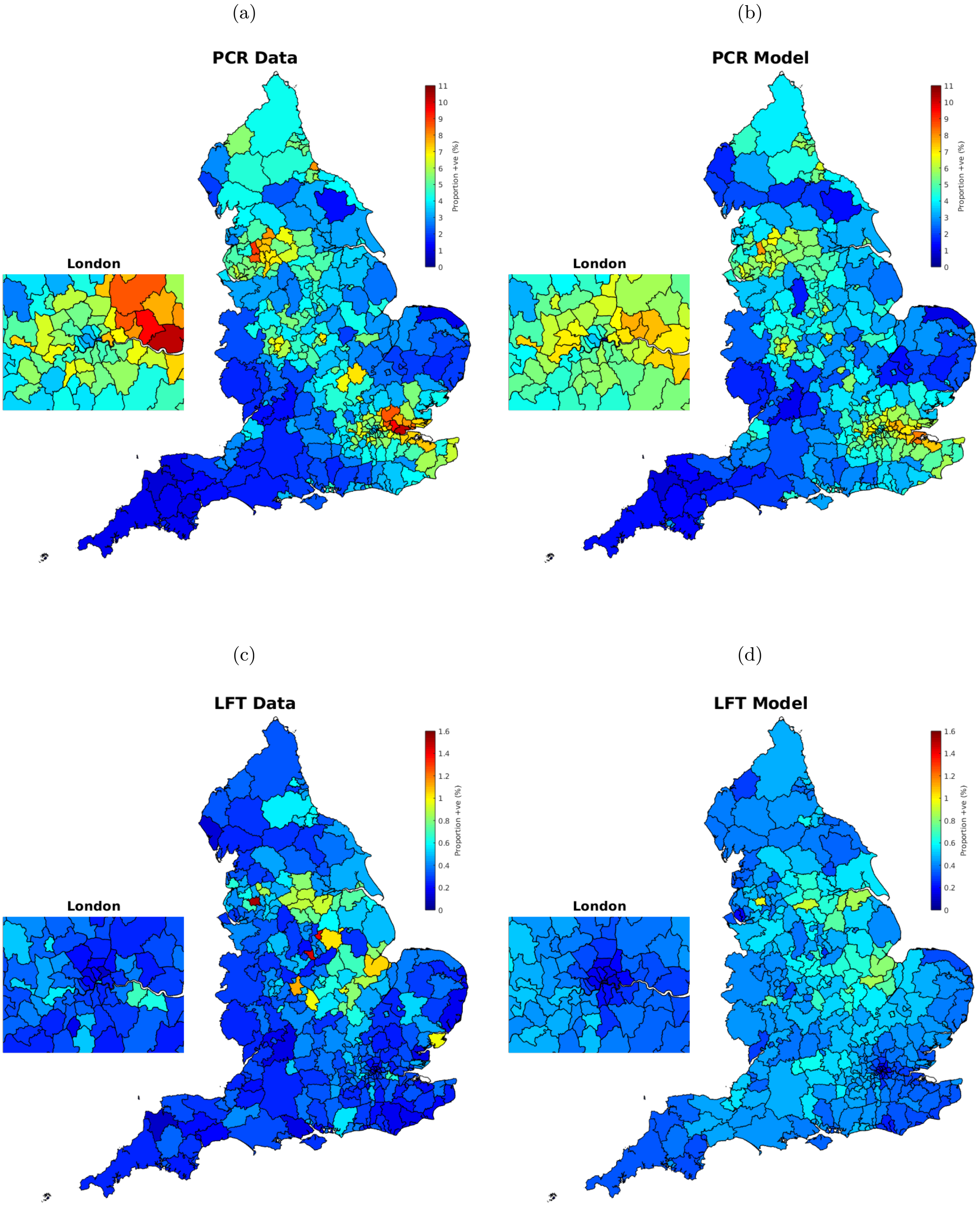


Supplementary Figure 16: **Comparing the fitted model against testing data by LTLA.** Each LTLA is shaded to signify (a) the proportion of 11-16 year olds and (b) the proportion of modelled pupils from the fitted model who have tested positive to a PCR test (excluding confirmatory PCR tests) from 1st September 2020 until 23rd May 2021, shaded according to the same colour range. Similarly, each LTLA is shaded to signify (c) the proportion of 11-16 year olds and (d) the proportion of modelled pupils from the fitted model who have tested positive to an LFT test from 8th March 2021 until 23rd May 2021, shaded according to the same colour range. We obtained our model results from 100 simulations of 2979 secondary schools, each with a distinct parameter set sample from the posterior distribution.

## Supplementary Text 7 Comparing the model against absences data

We compared the level of absences obtained from the model against COVID-19 related absences recorded in Department for Education: Education Setting Status data. While the model is not fitted to this data explicitly, the close contact group sizes and isolation policies of modelled schools should result in similar levels of absences. Accordingly, modelled absences matched closely with observed data nationally (Supplementary Figure 17). The model matched PCR testing data relatively closely at a regional level (Supplementary Figure 18), though it underestimated absences in London and the South East in November-December 2020, underestimated absences in the North West in May 2021, and overestimated absences in the South West in May 2021.

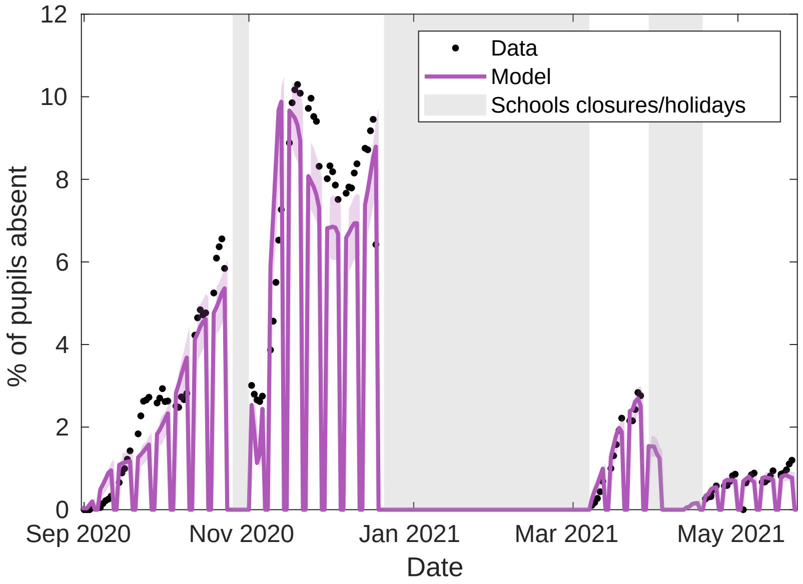


Supplementary Figure 17: **Comparing modelled absences against within-school COVID-19 related absences data**. The time-series of the modelled percentage of pupils absences (purple line) is compared to the observed percentage of pupils absent due to a confirmed case or a suspected case of COVID-19, or due to being told to isolate due to potential contact with a case of COVID-19 from inside their educational setting, for the 2979 secondary schools that recorded this data. Model results were obtained from 100 simulations of 2979 secondary schools, each with a distinct parameter set sample from the posterior distribution. Solid lines correspond to mean temporal profiles and shaded intervals represent 95% prediction intervals.

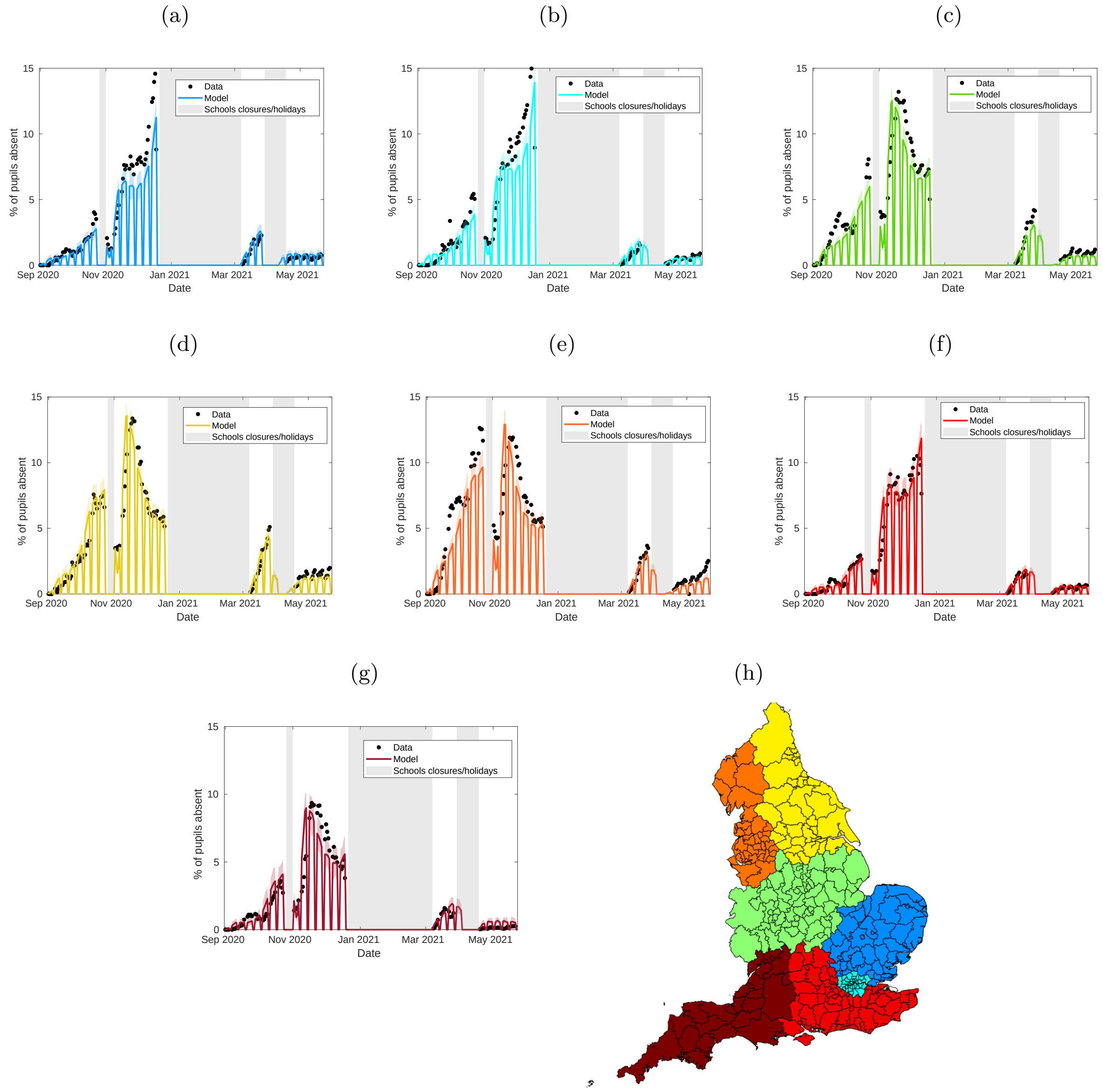


Supplementary Figure 18: **Comparing the fitted model against within-school COVID-19 related absences data by region.** The above plots compare modelled percentage of pupils absences compared to the observed percentage of pupils absent due to a confirmed case or a suspected case of COVID-19, or due to being told to isolate due to potential contact with a case of COVID-19 from inside their educational setting, for the 2979 secondary schools that recorded this data., for (a) East of England, (b) Greater London, (c), the Midlands, (d) the North-East, (e), the North-West, (f) the South-East, and (g) the South-West, with (h) showing the LTLAs that aggregated together formed each region, with colours corresponding to the time-series plots. We obtained these results from 100 simulations of 2979 secondary schools, each with a distinct parameter set sample from the posterior distribution. In all panels, solid lines correspond to mean temporal profiles and shaded ribbons represent 95% prediction intervals.

# Supplementary Text 8 Assuming greater relative levels of within-year and between-year mixing

This section contains analogous figures to Figures 2-4 from the main paper, under alternative within-school mixing assumptions. In the main analyses presented in the main body of the paper, we assumed that pupils interacted with their close contacts at a much higher rate than other members of their year group, and where year group bubbles have been in general very effective, setting $\alpha_1 = 0.1$ and $\alpha_2 = 0.01$. However, a precise understanding of current levels of within-school mixing remains unclear. While previous studies undertaken prior to the COVID-19 pandemic have attempted to record contact mixing patterns within schools[27–29], the implementation of rigid social distancing measures within schools mean that such studies are not of direct use in the context of COVID-19. The CoMix study has surveyed social contacts in the UK during the COVID-19 pandemic and has been used to infer age-dependent mixing matrices[30], though is not directly informative of contact structure within schools specifically. Accordingly, we assessed the sensitivity of of our results to these mixing assumptions.

To do so, we also fitted our model under the assumption that pupils within their year-group mix randomly, setting $\alpha_1 = 1$ and that there is a much higher-level of interaction between year groups, setting $\alpha_2 = 0.1$. It is important to note that the differing mixing assumptions between this section and the main paper only effects *who* an infected pupil will infect, rather than *how many*. These results should not be interpreted as justifying a relaxation of pre-existing within-school distancing measures, as doing so would not only change the mixing patterns within a school, but would also likely increase the number of infectious contacts pupils make.

Assuming $\alpha_1 = 1, \alpha_2 = 0.1$, we fitted the model via the ABC-SMC procedure outlined in Supplementary Text 5. The fitted model matches well to both temporal PCR and LFT positivity data (Supplementary Figures 19a and 19b), and the fits obtained to the to the distribution of peak confirmed cases from September-December 2020 and from March-May 2021 were qualitatively similar to the fitted model in the main analysis (Supplementary Figures 19c and 19d).

Qualitatively similar trends emerged in the estimates of within-school transmission and $R_{school}$, despite the differing assumptions regarding within school mixing (Supplementary Figure 20). The proportion of cases occurring within school increased through time, accounting for 42% (95% prediction interval: 23-60%) of all new infections in the September-October half term, 69%: (95% prediction interval: 59-78%) of all new infections in the November-December half term, and 74% (95% prediction interval: 65-82%) of infections since schools reopened from 8th March 2021 until 23rd May 2021.

As in the main analyses, the introduction of mass testing alongside isolation of close contacts substantially reduced incidence from 8th March-23rd May 2021 compared to a policy of isolation of close contacts only, and kept $R_{school}$ below 1 over the same period. Under these alternative mixing assumptions, we obtained qualitatively similar $R_{school}$ values to the $R_{school}$ values obtained for their counterparts in the main analysis. As in the main analysis, a strategy of mass testing alone, or a strategy of serial contact testing alongside mass testing led to substantially lower levels of absences than either strategy involving the isolation of close contacts (Supplementary Figure 21).

These results show that, once the model is calibrated to fit the available data, the relative levels of mixing assumed within schools has little bearing on the impact of control measures. An understanding of whether this holds in general, and a deeper understanding of the interplay between contact network structure within schools and the success of COVID-19 control measures, would be an important contribution going forward.

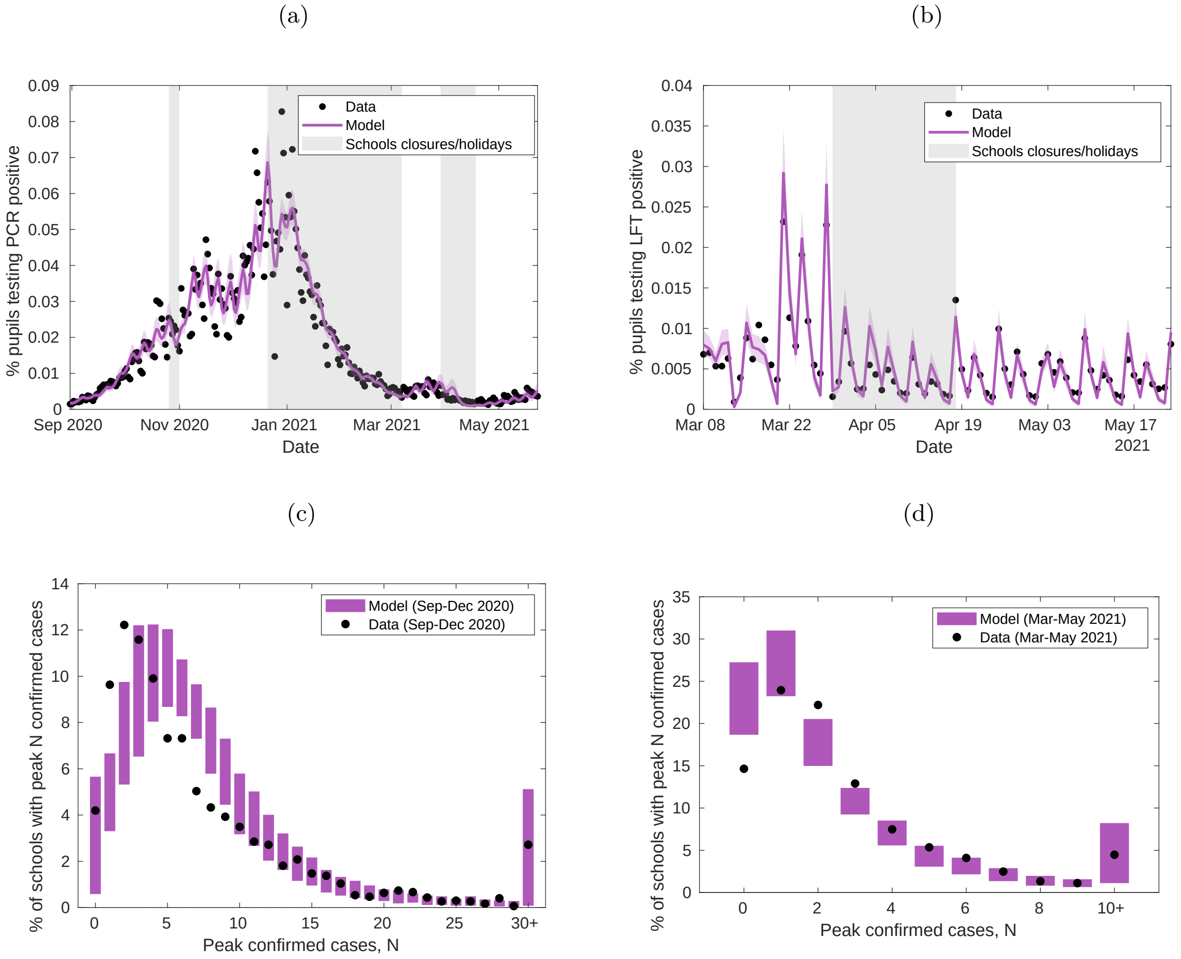


Supplementary Figure 19: **Fitting the model to testing and secondary school absences data, under different within-school mixing assumptions.** Under the alternative within-school mixing assumptions $\alpha_1 = 1$, $\alpha_2 = 0.1$, the stochastic individual-based model is fitted to (a) the percentage of 11-16 year olds who test PCR positive (excluding confirmatory PCR tests) each day from 1st September 2020 to 23rd May 2021, (b) the percentage of 11-16 year olds who test LFT positive each day from 8th March 2021 to 23rd May 2021. Shaded intervals around mean model traces (solid lines) represent 95% prediction intervals in all plots, while shaded grey regions represent time periods when schools were not fully open (either due to closures or school holidays). The model is also fitted to (c) the distribution of peak number of confirmed COVID-19 absences in secondary schools from 1st September 2020 to 18th December 2020, and (d) the distribution of peak number of confirmed COVID-19 absences in secondary schools from 8th March 2021 to 23rd May 2021. Filled circles denote the data and shaded blocks the 95% prediction interval estimated from the model. The model provides a reasonable fit to both distributions, though it overestimates the proportion of schools with a low peak number of confirmed cases in (d). The plots above show the mean values obtained from 100 simulations of 2979 secondary schools, each with a distinct parameter set sample from the posterior distribution.

(a) (b)

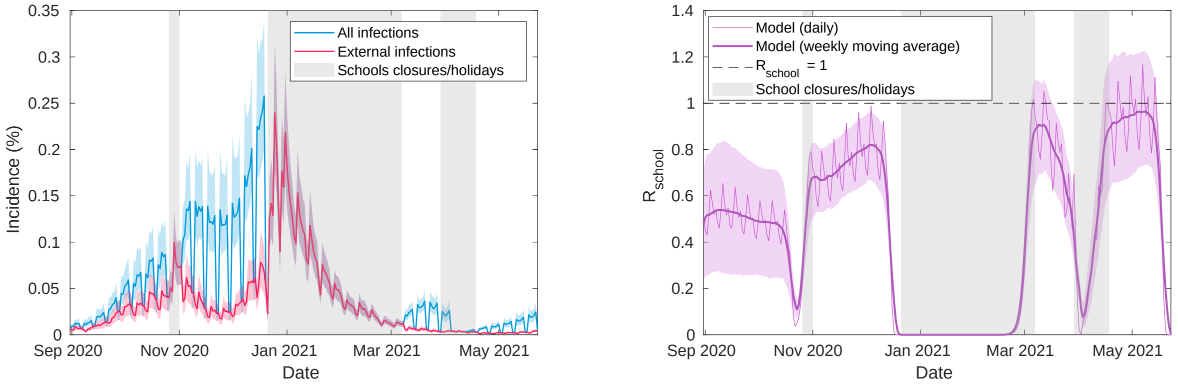


Supplementary Figure 20: **Incidence and $R_{school}$ from the fitted model under different within-school mixing assumptions.** For the model fitted under the alternative within-school mixing assumptions $\alpha_1 = 1$, $\alpha_2 = 0.1$, we display time-series of (a) total incidence among pupils (blue) alongside incidence occurring through external (non-school) infections (red), and (b) $R_{school}$ through time (thin line) alongside its seven-day moving average (thick line). The plots above were obtained from 100 simulations of 2979 secondary schools, each with a distinct parameter set sample from the posterior distribution. In all panels, solid lines correspond to mean temporal profiles, shaded ribbons represent 95% prediction intervals in all plots, while shaded grey regions represent time periods when schools were not fully reopen (either due to closures or school holidays).

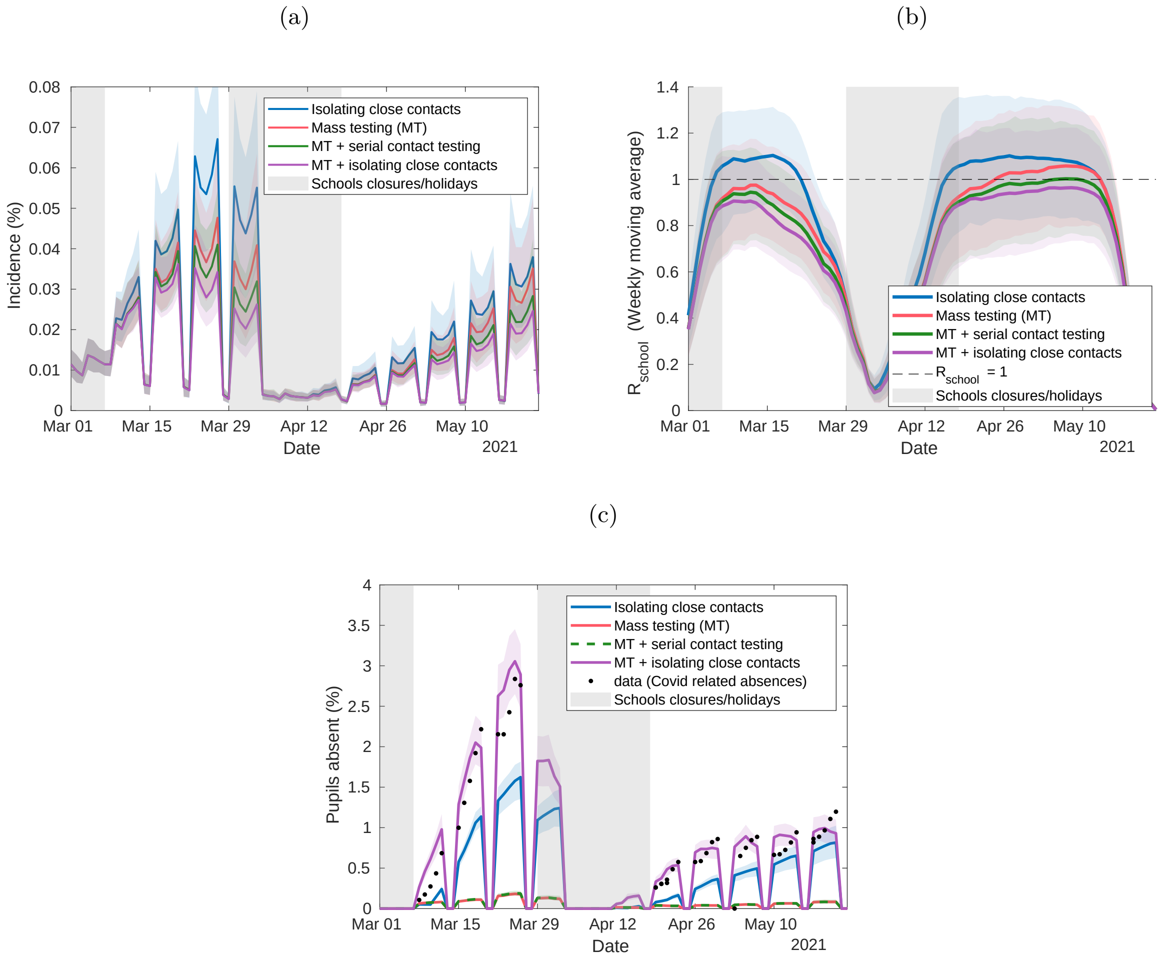


Supplementary Figure 21: **Quantifying the impact of LFTs on transmission and absences, and the potential impact of alternative strategies, under different within-school mixing assumptions.** Time-series under different intervention strategies for the model fitted under the alternative within-school mixing assumptions $\alpha_1 = 1$, $\alpha_2 = 0.1$ of (a) incidence among pupils, (b) $R_{school}$ within secondary schools, and (c) the percentage of pupils absent. We compare a policy of twice weekly mass testing and isolating close contacts (purple) to a strategy of isolating close contacts only (blue), twice weekly mass testing only (red), and twice weekly mass testing alongside serial contact testing (green). The plots above show the mean values obtained from 100 simulations of 2979 secondary schools, each with a distinct parameter set sample from the posterior distribution. In all panels, solid lines correspond to the mean estimate, shaded intervals represent 95% prediction intervals, while shaded grey regions represent time periods when schools were not fully reopen (either due to closures or school holidays). The data in panel (c) consists of the number of absences due to a confirmed case or a suspected case of COVID-19, and absences arising as a result of students told to isolate due to potential contact with a case of COVID-19 from inside their educational setting. This is taken from the 2979 secondary schools that recorded this data.

## References


[1] UK Government. Guidance: COVID-19 testing data: methodology note; 2021. Available from: https://www.gov.uk/government/publications/coronavirus-covid-19-testing-data-methodology/covid-19-testing-data-methodology-note.

[2] Office for National Statistics. Dataset: Estimates of the population for the UK, England and Wales, Scotland and Northern Ireland; 2021. Available from: https://www.ons.gov.uk/peoplepopulationandcommunity/populationandmigration/populationestimates/datasets/populationestimatesforukenglandandwalesscotlandandnorthernireland.

[3] UK Government: Prime Minister's Office. Educational setting status; 2020. Available from: https://form.education.gov.uk/service/educational-setting-status.

[4] Office for National Statistics. Coronavirus (COVID-19) Infection Survey pilot: England, Wales and Northern Ireland, 25 September 2020; 2020. Available from: https://www.ons.gov.uk/peoplepopulationandcommunity/healthandsocialcare/conditionsanddiseases/bulletins/coronaviruscovid19infectionsurveypilot/englandwalesandnorthernireland25september2020.

[5] Leng T, Hill EM, Thompson RN, Tildesley MJ, Keeling MJ, Dyson L. Assessing the impact of secondary school reopening strategies on within-school COVID-19 transmission and absences: a modelling study. medRxiv. 2021:2021.02.11.21251587. Available from: http://medrxiv.org/content/early/2021/02/12/2021.02.11.21251587.abstract.

[6] Hart WS, Maini PK, Thompson RN. High infectiousness immediately before COVID-19 symptom onset highlights the importance of continued contact tracing. Elife. 2021;10:e65534.

[7] Ferretti L, Ledda A, Wymant C, Zhao L, Ledda V, Abeler- Dorner L, et al. The timing of COVID-19 transmission. medRxiv. 2020:2020.09.04.20188516. Available from: http://medrxiv.org/content/early/2020/09/16/2020.09.04.20188516.abstract.

[8] Lauer SA, Grantz KH, Bi Q, Jones FK, Zheng Q, Meredith HR, et al. The Incubation Period of Coronavirus Disease 2019 (COVID-19) From Publicly Reported Confirmed Cases: Estimation and Application. Annals of Internal Medicine. 2020 3.

[9] Davies NG, Klepac P, Liu Y, Prem K, Jit M, CMMID COVID-19 working group, et al. Age-dependent effects in the transmission and control of COVID-19 epidemics. Nat Med. 2020 aug;26(8):1205-11. Available from: http://www.nature.com/articles/s41591-020-0962-9.

[10] McEvoy D, McAloon CG, Collins AB, Hunt K, Butler F, Byrne AW, et al. The relative infectiousness of asymptomatic SARS-CoV-2 infected persons compared with symptomatic individuals: A rapid scoping review. medRxiv. 2020:2020.07.30.20165084. Available from: http://medrxiv.org/content/early/2020/08/01/2020.07.30.20165084.abstract.

[11] Buitrago-Garcia D, Egli-Gany D, Counotte MJ, Hossmann S, Imeri H, Ipekci AM, et al. Occurrence and transmission potential of asymptomatic and presymptomatic SARS-CoV-2 infections: A living systematic review and meta-analysis. PLOS Med. 2020 sep;17(9):e1003346.

[12] Davies NG, Abbott S, Barnard RC, Jarvis CI, Kucharski AJ, Munday JD, et al. Estimated transmissibility and impact of SARS-CoV-2 lineage B. 1.1. 7 in England. Science. 2021;372(6538).

[13] Volz E, Mishra S, Chand M, Barrett JC, Johnson R, Geidelberg L, et al. Assessing transmissibility of SARS-CoV-2 lineage B. 1.1. 7 in England. Nature. 2021;593(7858):266-9.

[14] Graham MS, Sudre CH, May A, Antonelli M, Murray B, Varsavsky T, et al. Changes in symptomatology, reinfection, and transmissibility associated with the SARS-CoV-2 variant B. 1.1. 7: an ecological study. The Lancet Public Health. 2021;6(5):e335-45.

[15] Office for National Statistics. COVID-19 Schools Infection Survey Round 2, England: December 2020; 2021. Available from: https://www.ons.gov.uk/peoplepopulationandcommunity/healthandsocialcare/conditionsanddiseases/bulletins/covid19schoolsinfectionsurveyround2england/december2020.

[16] UK Government: Teaching Blog. How schools are managing bubbles effectively; 2021. Available from: https://teaching.blog.gov.uk/2020/09/28/how-schools-are-managing-bubbles-effectively/.

[17] UK Government. Guidance for schools: coronavirus (COVID-19); 2020. Available from: https://www.gov.uk/government/collections/guidance-for-schools-coronavirus-covid-19.

[18] Public Health England. Stay at home: guidance for households with possible or confirmed coronavirus (COVID-19) infection.; 2021. Available from: https://www.gov.uk/government/publications/covid-19-stay-at-home-guidance/stay-at-home-guidance-for-households-with-possible-coronavirus-covid-19-infection.

[19] Department of Health and Social Care. NHS test and trace: how it works.; 2021. Available from: https://www.gov.uk/guidance/nhs-test-and-trace-how-it-works.

[20] Hellewell J, Russell TW, Beale R, Kelly G, Houlihan C, Nastouli E, et al. Estimating the effectiveness of routine asymptomatic PCR testing at different frequencies for the detection of SARS-CoV-2 infections. BMC Med. 2021 dec;19:106. Available from: https://bmcmedicine.biomedcentral.com/articles/10.1186/

s12916-021-01982-x.

[21] Office for National Statistics. Coronavirus (COVID-19) Infection Survey: antibody data for the UK, January 2021; 2021. Available from: https://www.ons.gov.uk/peoplepopulationandcommunity/healthandsocialcare/conditionsanddiseases/articles/coronaviruscovid19infectionsinthecommunityinengland/antibodydatafortheukjanuary2021.

[22] Funk S, Munblit D, Flasche S. LFD mass testing in English schools: additional evidence of high test specificity. CMMID Repository. 2021. Available from: https://cmmid.github.io/topics/covid19/mass-testing-schools.html.

[23] Department of Health and Social Care. Lateral flow device specificity in phase 4 (post-marketing) surveillance; 2021. Available from: https://www.gov.uk/government/publications/lateral-flow-device-specificity-in-phase-4-post-marketing-surveillance.

[24] Toni T, Welch D, Strelkowa N, Ipsen A, Stumpf MPH. Approximate Bayesian computation scheme for parameter inference and model selection in dynamical systems. J R Soc Interface. 2009 feb;6(31):187-202.

[25] Brooks-Pollock E, Roberts GO, Keeling MJ. A dynamic model of bovine tuberculosis spread and control in Great Britain. Nature. 2014;511(7508):228-31.

[26] Volz E, Mishra S, Chand M, Barrett JC, Johnson R, Geidelberg L, et al. Transmission of SARS-CoV-2 Lineage B.1.1.7 in England: Insights from linking epidemiological and genetic data. medRxiv. 2021:2020.12.30.20249034. Available from: http://medrxiv.org/content/early/2021/01/04/2020.12.30.20249034.1.abstract.

[27] Salathé M, Kazandjieva M, Lee JW, Levis P, Feldman MW, Jones JH. A high-resolution human contact network for infectious disease transmission. Proceedings of the National Academy of Sciences. 2010;107(51):22020-5.

[28] Eames KT, Tilston NL, Edmunds WJ. The impact of school holidays on the social mixing patterns of school children. Epidemics. 2011;3(2):103-8.

[29] Conlan AJ, Eames KT, Gage JA, von Kirchbach JC, Ross JV, Saenz RA, et al. Measuring social networks in British primary schools through scientific engagement. Proceedings of the Royal Society B: Biological Sciences. 2011;278(1711):1467-75.

[30] Jarvis CI, Van Zandvoort K, Gimma A, Prem K, CMMID COVID-19 working group, Klepac P, et al. Quantifying the impact of physical distance measures on the transmission of COVID-19 in the UK. BMC Med. 2020 dec;18:124. Available from: https://bmcmedicine.biomedcentral.com/articles/10.1186/s12916-020-01597-8.
