# Supplementary material for: Quantifying pupil-to-pupil SARS-CoV-2 transmission and the impact of lateral flow testing in English secondary schools
Source: Nat Commun. 2022 Mar 1;13:1106. doi: 10.1038/s41467-022-28731-9 (PMC8888696; doi:10.1038/s41467-022-28731-9)
Supplement: Supplementary file 2 — Reporting Summary [file 41467_2022_28731_MOESM2_ESM.pdf]

nature portfolio

Corresponding author(s): Trystan Leng

Last updated by author(s): Jan 24, 2021

Reporting Summary

Nature Portfolio wishes to improve the reproducibility of the work that we publish. This form provides structure for consistency and transparency in reporting. For further information on Nature Portfolio policies, see our Editorial Policies and the Editorial Policy Checklist.

Statistics

For all statistical analyses, confirm that the following items are present in the figure legend, table legend, main text, or Methods section.

n/a	Confirmed	
☐	☒	The exact sample size (n) for each experimental group/condition, given as a discrete number and unit of measurement
☒	☐	A statement on whether measurements were taken from distinct samples or whether the same sample was measured repeatedly
☒	☐	The statistical test(s) used AND whether they are one- or two-sided<br>*Only common tests should be described solely by name; describe more complex techniques in the Methods section.*
☒	☐	A description of all covariates tested
☒	☐	A description of any assumptions or corrections, such as tests of normality and adjustment for multiple comparisons
☐	☒	A full description of the statistical parameters including central tendency (e.g. means) or other basic estimates (e.g. regression coefficient) AND variation (e.g. standard deviation) or associated estimates of uncertainty (e.g. confidence intervals)
☒	☐	For null hypothesis testing, the test statistic (e.g. F, t, r) with confidence intervals, effect sizes, degrees of freedom and P value noted<br>*Give P values as exact values whenever suitable.*
☐	☒	For Bayesian analysis, information on the choice of priors and Markov chain Monte Carlo settings
☒	☐	For hierarchical and complex designs, identification of the appropriate level for tests and full reporting of outcomes
☒	☐	Estimates of effect sizes (e.g. Cohen's d, Pearson's r), indicating how they were calculated

Our web collection on statistics for biologists contains articles on many of the points above.

Software and code

Policy information about availability of computer code

Data collection	No software was used for data collection.
Data analysis	Code for the model and model fitting is available at: https://github.com/tsleng93/SchoolReopeningStrategies/FittedModel/

For manuscripts utilizing custom algorithms or software that are central to the research but not yet described in published literature, software must be made available to editors and reviewers. We strongly encourage code deposition in a community repository (e.g. GitHub). See the Nature Portfolio guidelines for submitting code & software for further information.

Data

Policy information about availability of data

All manuscripts must include a data availability statement. This statement should provide the following information, where applicable:

- Accession codes, unique identifiers, or web links for publicly available datasets
- A description of any restrictions on data availability
- For clinical datasets or third party data, please ensure that the statement adheres to our policy

Data from the Department for Education Educational Settings database were supplied after anonymisation under strict data protection protocols agreed between the University of Warwick and the Department for Education in the UK. The ethics of the use of these data for these purposes was agreed by the Department for Education with the Government's SPI-M(O)/SAGE committees.

Public Health England (PHE) collected data in a centralised database, which included details on the type of test and results. PHE provided anonymised data to contributors of the Scientific Pandemic Influenza Group on Modelling (SPI-M) as part of the COVID-19 response under a data sharing agreement between PHE and the authors' institutions.

Field-specific reporting

Please select the one below that is the best fit for your research. If you are not sure, read the appropriate sections before making your selection.

- [x] Life sciences
- [ ] Behavioural & social sciences
- [ ] Ecological, evolutionary & environmental sciences

For a reference copy of the document with all sections, see nature.com/documents/nr-reporting-summary-flat.pdf

Life sciences study design

All studies must disclose on these points even when the disclosure is negative.

Sample size	2979 secondary schools - i.e. all secondary schools who recorded Department for Education: Educational Setting Status data between 31st August 2020 and 21st May 2021
Data exclusions	None
Replication	The model fitting and simulations to generate the model results were seeded with known random seeds with known software version (Matlab 2021a), in order to be able to replicate model fits and model results exactly. Doing so we could successfully replicate the modelling results in the main text and supplementary text, and we were able to reproduce the stages of the model fitting algorithm.
Randomization	As this was a modelling study, there were no participants to allocate into experimental groups. As such, randomization was not applicable to this study. All schools from the data were included within the model data set.
Blinding	Our results were on the basis of comparing simulations, rather than comparing data from separate data groups. Accordingly, schools/ participants were not allocated into separate groups, and as such, blinding was not applicable to our modelling study.

Reporting for specific materials, systems and methods

We require information from authors about some types of materials, experimental systems and methods used in many studies. Here, indicate whether each material, system or method listed is relevant to your study. If you are not sure if a list item applies to your research, read the appropriate section before selecting a response.

Materials & experimental systems

n/a	Involved in the study	
x		Antibodies
x		Eukaryotic cell lines
x		Palaeontology and archaeology
x		Animals and other organisms
x		Human research participants
x		Clinical data
x		Dual use research of concern

Methods

n/a	Involved in the study	
x		ChIP-seq
x		Flow cytometry
x		MRI-based neuroimaging
